# *Forsythiae Fructus*: A Review on its Phytochemistry, Quality Control, Pharmacology and Pharmacokinetics

**DOI:** 10.3390/molecules22091466

**Published:** 2017-09-04

**Authors:** Zhanglu Dong, Xianyuan Lu, Xueli Tong, Yaqian Dong, Lan Tang, Menghua Liu

**Affiliations:** Guangdong Provincial Key Laboratory of New Drug Screening, School of Pharmaceutical Sciences, Southern Medical University, Guangzhou 510515, China; m17817185212@163.com (Z.D.); luxianyuan_@163.com (X.L.); xueli_tong@126.com (X.T.); 13246808042@163.com (Y.D.)

**Keywords:** *Forsythiae Fructus*, phytochemistry, quality control, pharmacology, pharmacokinetics

## Abstract

*Forsythiae Fructus*, as a traditional Chinese medicine, has been widely used both as a single herb and in compound prescriptions in Asia, mainly due to its heat-clearing and detoxifying effects. Modern pharmacology has proved *Forsythiae Fructus* possesses various therapeutic effects, both in vitro and in vivo, such as anti-inflammatory, antibacterial and antiviral activities. Up to now, three hundred and twenty-one compounds have been identified and sensitive analytical methods have been established for its quality control. Recently, the pharmacokinetics of *Forsythiae Fructus* and its bioactive compounds have been reported, providing valuable information for its clinical application. Therefore, this systematic review focused on the newest scientific reports on *Forsythiae Fructus* and extensively summarizes its phytochemistry, pharmacology, pharmacokinetics and standardization procedures, especially the difference between the two applied types—unripe *Forsythiae Fructus* and ripe *Forsythiae Fructus*—in the hope of providing a helpful reference and guide for its clinical applications and further studies.

## 1. Introduction

*Forsythiae Fructus*, the dried fruit of *Forsythia suspensa* (family Oleaceae), known as lianqiao in China, was first recorded in *Shennong Bencao Jing*, a prestigious monograph on traditional Chinese medicine (TCM) [1], and subsequently listed in the pharmacopoeias of the People’s Republic of China, Japan and Korea [2,3,4]. It has been used as a heat-clearing and detoxifying TCM for the treatment of infectious diseases, such as acute nephritis, erysipelas and ulcers, for over 2000 years [5,6]. Modern pharmacological studies have confirmed that *Forsythiae Fructus* possesses anti-inflammatory, antioxidant, antiviral, antivomiting and antitumor activities, as well as hepatoprotective, neuroprotective and cardiovascular protective effects [7,8,9,10,11,12]. Nowadays, more than forty Chinese medicinal preparations containing *Forsythiae Fructus* are included in the Chinese Pharmacopoeia, Volume I [2]. For example, *Forsythiae Fructus* is used as a principal drug in Yinqiao Jiedu tablet exerting effects of expelling wind, relieving the exterior, clearing heat and detoxifying [2].

In the clinic two types of *Forsythiae Fructus* are used, namely the unripe *Forsythiae Fructus* (Qing qiao, UFF) and ripe *Forsythiae Fructus* (Lao qiao, RFF). Due to the different harvest times, they are distinguished as UFF and RFF collected at early September and October, respectively [6]. Although both of them have been listed as *Forsythiae Fructus* in the Chinese Pharmacopoeia, previous studies have found that the harvest time could affect the qualitative profile and relative contents of compounds in *Forsythiae Fructus*, which might further influence its pharmacological activities. For instance, Jia et al. [6] found a higher antioxidant activity for UFF than for RFF, but no significant difference in antibacterial activities was shown, indicating the differences between UFF and RFF should be considered for their clinical efficacies.

Up to now, a large number of studies focusing on the chemical compounds, pharmacology and quantitative analysis of *Forsythiae Fructus* have been published. In 2012, a mini review [13] naming chemical constituents of plants from the genus *Forsythia* reported only one hundred and twenty-one chemical constituents in *Forsythiae Fructus*, which was much less than those we summarize herein (three hundred and twenty-one compounds). New pharmacological activities and quality control methods have been discovered, so a systematic and updated review is very necessary, as well as a comprehensive comparison between UFF and RFF. Therefore, this review aims to extensively summarize the phytochemistry, quality control data, pharmacology and pharmacokinetics of *Forsythiae Fructus*, thus providing evidence for further research and clinical applications of this plant.

## 2. Phytochemistry

With the analysis technologies of nuclear magnetic resonance (NMR), liquid chromatography-mass spectrometry (LC-MS), gas chromatography-mass spectrometry (GC-MS) and infrared spectroscopy (IR), a total of three hundred and twenty-one compounds were identified from *Forsythiae Fructus*, including fifty-one phenylethanoid glycosides, fifty lignans, nineteen aliphatic alcohols with the C6-C2 skeleton, two iridoids, nineteen diterpenoids, twenty-seven triterpenoids, six sterols, nineteen flavonoids, fifty-two volatiles, seven alkaloids, twenty-eight organic acids, six amino acids, nine sugar derivatives, two allylbenzene glycosides and twenty-four others. Most of them were not mentioned whether obtained from UFF or RFF. The detailed information for these compounds is summarized in Table 1.

### 2.1. Phenylethanoid Glycosides

Phenylethanoid glycosides are the major bioactive constituents of *Forsythiae Fructus* with verified anti-inflammatory, antioxidant, antibacterial and antiviral effects [27,28,76,77]. Since forsythoside A (**1**) was reported by Endo et al. [20] in 1984, fifty-one phenylethanoid glycosides have been isolated from *Forsythiae Fructus* and their structures were shown in Figure 1. Except for (*R*)-suspensaside (**3**), (*S*)-suspensaside (**4**), (*S*)-suspensaside methyl ether (**5**), β-methoxyforsythoside E (**11**), acteoside (**16**), forsythoside B (**17**), forsythoside G (**18**), (*S*)-β-hydroxycalceolarioside C (**22**), (*R*)-β-hydroxycalceolarioside C (**23**), (S)-β-methoxycalceolarioside C (**24**), (*R*)-β-methoxycalceolarioside C (**25**), derhamnosyl suspensaside (**27**), β-methoxylacteoside (**28**), caffeoyl calceolarioside C (**29**), β-methoxyferruginoside B (**31**), β-methoxylipedoside A (**32**), suspensaside A isomer (**40**) and demethyl suspensaside A (**41**) tentatively identified by a HPLC/MS^n^ method [17,18,22], the remaining compounds were isolated from the 50%, 60%, 70%, 75% or 85% ethanol extract of *Forsythiae Fructus* and then comfirmed by NMR [11,15,16,21,23,24,25,26,27,28,30]. In addition, forsythoside A (**1**) is recommended as the marker compound for the quality control of this plant in the Chinese Pharmacopoeia [2].

### 2.2. Lignans

The lignans are another major bioactive constituents in *Forsythiae Fructus* and their structures are shown in Figure 2. They are mainly classified into six groups: seven dibenzylbutyrolactones (**52**–**58**), nineteen furofurans (**59**–**77**), four arylnaphthalenes (**78**–**81**), five benzylfurans (**82**–**87**), nine tetrahydrofurans (**88**–**91**,**95**–**100**) and one dibenzylbutane (benzenebutanoic acid, **34**). Structures of these compounds were confirmed by NMR after isolation from the methanol or 50% ethyl acetate extract of *Forsythiae Fructus*. Compounds, such as arctigenin (**52**), arctiin (**53**), matairesinoside (**54**), matairesinol (**55**), 2′,5′-dihydroxy-4′′-caffeoyl matairesinol (**56**), 3′,4′,5′-trihydroxy-3′′-methoxy-4′′-caffeoyl lignin (**57**), caffeoyl phillygenin (**61**), pinoresinol diglucoside (**71**), caffeoyl pinoresinol (**72**), 3′,4′,5′-trimethoxy-4′′-hydroxyllignan *O*-glucoside (**76**) and 3-furanone-2-(3-methoxy-4-hydroxy-phenyl)-4-veratryl (**97**) were tentatively identified by molecular weight and fragmentations by a HPLC-MS^n^ method [17,22]. Among these compounds, phillyrin (**60**) is also recommended as the marker compound for *Forsythiae Fructus* in the Chinese pharmacopoeia [2].

### 2.3. Aliphatic C6-C2 Alcohols

To date, eighteen natural alcohols with the C_6_-C_2_ skeletons have been isolated from *Forsythiae Fructus*, since rengyol (**103**), rengyolone (**110**) and rengyoxide (**111**) were first reported in 1984 by Endo et al. [20]. Subsequently, they identified isorengyol (**102**), rengyoside A (**106**) and rengyoside C (**107**) in 1987 and 1989 [29,40]. Compounds cornoside (**113**), forsythenside A (114), forsythenside B (**115**), forsythensides G-J (**118**–**120**), togerther with rengyolester (**105**) were obtained from 60%, 70% or 75% ethanol extract of *Forsythiae Fructus* [23,42,45], whereas suspenol (**104**), rengynic acid-1′-*O*-β-d-glucopyranoside (**109**) and forsythenside F (**116**) were isolated from methanol extract, aqueous extract and 50% acetone extract respectively [41,44,46]. The structures of these compounds are shown in Figure 3.

### 2.4. Iridoids, Diterpenoids and Triterpenoids

As shown in Figure 4, two iridoids (**121**–**122**), nineteen diterpenoids (**123**–**141**) and twenty-seven triterpenoids (**142**–**168**) have been confirmed in *Forsythiae Fructus*. Most of them were reported by Kuo et al. [38] in 2017. The triterpenoid fraction contains eleven tetracyclic triterpenoids (**142**–**152**) and sixteen pentacyclic triterpenoids (**136**–**151**). Compounds such as ocotillone (**142**), ocotillol monoacetate (**143**) and oleanolic acid (**153**) were obtained from the 70% ethanol extract of *Forsythiae Fructus* [49,52]. Rouf et al. [50] found two new triterpenoids, namely 3β-acetyl-20,25-epoxy-dammarane-24α-ol (**145**) and 3β-acetyl-20,25-epoxydammarane-24β-ol (**146**) and confirmed their anti-inflammatory activities. Xue et al. [47] revealed dammar-24-en-3β-acetoxy-20-ol (**147**), 3β-acetoxy-20*S*,24*R*-dammarane-25-ene-24-hydroperoxy-20-ol (**149**) and 3-acetylisofouquierol (**152**) possessing strong anti-proliferative effect on MKN-45, BGC-823 and SGC-9701 cells in the 95% ethanol extract of *Forsythiae Fructus*.

### 2.5. Sterols

Six sterols, namely β-sitosterol (**169**), daucosterol (**170**), taraxasterol acetate (**171**), stigmasterol (**172**), *ψ*-taraxasterol (**173**) and (6′-*O*-palmitoyl)-sitosterol-3-*O*-β-d-glucoside (**174**), have been isolated from *Forsythiae Fructus* and identified by ^1^H- and ^13^C-NMR [48,49,56,57]. Their structures are shown in Figure 5.

### 2.6. Flavonoids

**Figure 6:**
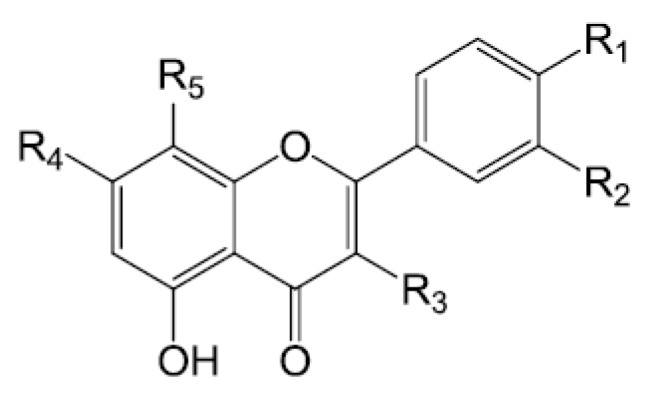
Chemical structures of flavonoids in *Forsythiae Fructus.*

Flavonols, represented by quercetin (**177**) and its derivatives (**178**–**179**), are the main types of flavonoids identified in *Forsythiae Fructus*. Forsythoneosides A−D (**190**–**193**), four unusual condensation products of flavonoids and phenylethanoid glycosides isolated from the 75% ethanolic extract, displayed neuroprotective effects on rotenone-injured PC12 cells [11]. One rutin derivative (**176**), two quercetin derivatives (**178**–**179**) and two kaempferol derivatives (**183**–**184**) were extracted by 50% aqueous methanol and identified by HPLC-MS, but the exact attachment positions of the saccharides were unknown [22]. In addition, wogonin-7-*O*-glcoside (**187**) and baicalin (**188**), belonging to flavones, were also found in *Forsythiae Fructus* [58,60]. Their chemical structures are presented in Figure 6.

### 2.7. Volatiles

*Forsythiae Fructus* is also rich in volatiles. A total of fifty-two compounds with anti-inflammatory, anti-oxidant and antimicrobial effects were identified in the oil by GC-MS [61,62,63,64,65,66]. β-pinene (**195**, 45.88%), myrtenol (**196**, 13.86%), (+)-α-pinene (**197**, 13.09%), (−)-*trans*-pinocarveol (**198**, 7.34%), sabinene (**199**, 6.64%) and pinocarvone (**200**, 4.13%) were the major volatiles of *Forsythiae Fructus* [61]. Zhai et al. [63] compared five methods, including ionic liquid microwave extraction, hydrodistillation, microwave hydrodistillation, solvent-free microwave extraction and improved solvent-free microwave extraction to extract volatiles, but no significant difference in the oil composition was found. Jiao et al. [64] developed an enzyme-assisted microwave hydro-distillation method, which reached a maximum extraction yield of 3.27%.

### 2.8. Alkaloids

Alkaloids represent a relatively small class of compounds in *Forsythiae Fructus*. To date, seven alkaloids, namely rutaecarpine (**246**), suspensine A (**247**), (−)-egenine (**248**), (−)-7′-*O*-methylegenine (**249**), (−)-bicuculline (**250**), bis-2-(4-aminophenyl)ethyl-β-d-glucopyranoside (**251**) and choline (**252**) were obtained from the ethanolic extract of *Forsythiae Fructus* [6,57,67,68]. Their chemical structures are presented in Figure 7.

### 2.9. Others

Moreover, other compounds, including twenty-eight organic acids (**253**–**280**), six amino acids (**281**–**286**), nine sugar derivatives (**287**–**295**), two allylbenzene glycosides (**296**–**297**) and some miscellaneous compounds (**298**–**321**) were also obtained from *Forsythiae Fructus* [6,14,18,23,30,36,45,48,56,58,60,69,70,71,72,73,74]. Their structures are shown in Figure 8.

## 3. Quality Control

Quality control is very important for the use of TCMs. Many rapid, sensitive and stable technologies, such as HPLC–ESI-MS/MS, LC–MS/MS and HPLC-ESI-MS have been applied for quantitative analysis of *Forsythiae Fructus* [18,31,58,66,78,79,80,81,82,83,84,85,86,87,88,89,90,91]. A total of twenty-nine compounds: forsythoside A (**1**), (*R*)-suspensaside (**3**), (*S*)-suspensaside (**4**), (*S*)-suspensaside methyl ether (**5**), forsythoside E (**10**), forsythoside B (**17**), suspensaside A (**39**), arctigenin (**52**), matairesinol-4′-*O*-glucoside (**58**), phillygenin (**59**), forsythin (**60**), phillyrin (**60**), (+)-epipinoresinol (**62**), (+)-epi-pinoresinol-4′-*O*-β-d-glucoside (**64**), pinoresinol (**68**), (+)-pinoresinol-β-d-glucoside (**69**), rutin (**175**), quercetin (**177**), hyperin (**182**), baicalin (**188**), hesperidin (**189**), chlorogenic acid (**258**) anchoic acid (**259**), 4-hydroxy-4-isopropylcyclohex-1-ene carboxylic acid (**260**), *p*-coumaric acid (**261**) *p*-hydroxy-benzoic acid (**264**), cafferic acid (**268**), *p*-hydroxyphenylethanol (**314**) and *p*-hydroxybenzyl alcohol (**315**) have been quantified by HPLC or HPLC-MS by different research groups [18,58,78,79,80,81,82,83,84,85,86,87,88,89,90]. The volatile substances, such as β-pinene (**194**), camphene (**202**), myrcene (**203**), α-pinene (**212**), α-terpineol (**236**), *p*-cymene (**244**) and limonene (**245**) were detected by GC [66]. Interestingly, the contents of forsythoside A (**1**), phillygenin (**59**), phillyrin (**60**), (+)-epipinoresinol (**62**), (+)-epi-pinoresinol-4-*O*-β-d-glucoside (**64**), (+)-pinoresinol-β-d-glucoside (**69**) and rutin (**175**) were 0.85–15.71%, 0.02898–2.16%, 1.08–1.27%, 1.11–2.10%, 0.91–1.64%, 0.52–1.44% and 0.05–0.36%, respectively, in UFF and 0.02968–10.59%, 0.02148–2.5%, 0.08–0.54%, 0.16–0.64%, 0.22–0.58%, 0.12–0.48% and 0.0556–0.0583%, respectively, in RFF. Jia et al. [6] revealed that RFF contained much more forsythoside A, forsythoside C, rutin and phillyrin (5.07 times, 2.78 times, 2.62 times, 1.35 times, respectively) than UFF, whereas the amino acid content in the UFF was higher than that in the RFF. In addition, the volatile compounds of α-pinene and β-pinene were 0.102–0.337% and 0.342–1.024% in the UFF, respectively, which were higher than the levels in the RFF [66,91]. The harvest times could affect the contents of active compounds in *Forsythiae Fructus*, which should be considered when assessing their clinical efficacies. The quantitative analysis of *Forsythiae Fructus* are listed in Table 2.

## 4. Pharmacology

*Forsythiae Fructus* has long been used in China, Korea, Japan and other Southeast Asian countries because of its various pharmacological effects. The bioactivities of the active constituents of *Forsythiae Fructus*, including phenylethanoid glycosides, lignans and flavonoids, have been studied, but these constituents also exhibit new pharmacological activities. The pharmacological effects of this herb are listed in Table 3.

### 4.1. Anti-Inflammatory Effect

The anti-inflammatory effect of *Forsythiae Fructus* is its most common clinical use. According to Taiwan’s nationwide prescription database, *Forsythiae Fructus* has been listed in the top 10 most commonly used single herbs for the treatment of atopic dermatitis (15.9%), urticaria (11.49–13.4%) and acne (22.3%) [5,156,157]. Recently, numerous studies have found that ethanol, methanol and aqueous extracts of *Forsythiae Fructus* exhibited significant anti-inflammatory effects in vitro and in vivo [92,93,94,95,96,97,98]. In addition, its volatiles showed an anti-inflammatory effect in models of mouse ear-swelling, mouse celiac capillary permeability, rat paw-swelling, rat hind paw edema, oleic acid-stimulated acute lung injury and rat cotton pellet granuloma by inhibiting the release of prostaglandin 2 (PGE2), histamine and serotonin [99]. Forsythoside A (**1**), arctigenin (**52**), arctiin (**53**), matairesinol (**55**), phillyrin (**60**), forsythin (**60**), suspensine A (**247**), (−)-egenine (**248**), 7′-*O*-methylegenine (**249**) and (−)-bicuculline (**250**) were active compounds isolated from *Forsythiae Fructus* and exhibited anti-inflammatory effects [67,100,101,103,104,106,109]. Forsythoside A (**1**) decreased the levels of pro-inflammatory mediators, including tumor necrosis factor-α (TNF-α), interleukin-1β (IL-1β), nitric oxide (NO) and PGE2 in lipopolysaccharide (LPS)-stimulated BV2 microglia cells, RAW264.7 cells, human bronchial epithelial cells (BEAS-2B), acute liver injury mice and bursa of Fabricius of chicken, as well as in a mouse model of cigarette smoke-induced lung damage, through influencing the nuclear factor-κB (NF-κB), mitogen activated protein kinase (MAPK) and nuclear related factor 2/heme oxygenase 1(Nrf2/HO-1) signaling pathways [76,100,102,105,107,110]. Phillyrin (**60**) at 20 mg/kg showed an ameliorative effect on LPS-induced alveolar hemorrhage and neutrophil infiltration in lung injury mice by decreasing the production of TNF-α, IL-1β and interleukin-6 (IL-6) through MAPK and NF-κB signaling pathways [103]. 

Forsythin (**60**), a novel PDE4 inhibitor, inhibited the expression of PDE4 and production of NO, inducible nitric oxide synthase (iNOs), Toll-like receptor 4 (TRL4), TNF-α, IL-1β in LPS-induced lung injury mice, LPS-stimulated BV2 microglial cells and *Staphylococcus aureus*-induced monocyte-macrophages [94,95,99,111]. Arctiin (**53**) exhibited an anti-inflammatory effect in LPS-damaged macrophage cells by inhibiting the production of NO, PGE2, TNF-α, IL-1β, IL-6 and the expression of COX-2 [104]. Four alkaloids, namely suspensine A (**247**), (−)-egenine (**248**), 7′-*O*-methylegenine (**249**) and (−)-bicuculline (**250**), demonstrated an anti-inflammatory effect at a concentration of 10 μM by inhibiting the release of β-glucuronidase from polymorphonuclear leukocytes in the range of 34.8% to 39.6% [67]. In a word, the anti-inflammatory effects of *Forsythiae Fructus* and its constituents are closely related to the inhibition of pro-inflammatory mediators through activation of the Nrf2/HO-1 signaling pathway and downregulation of the NF-κB, JAK-STAT and p38 MAPK signaling pathways [101,104,107,110].

### 4.2. Antibacterial Effect

In vitro, the volatiles of *Forsythiae Fructus* exhibited good antibacterial effects against *S. pneumoniae*, *Escherichia coli* (*E. coli*), *Staphylococcus aureus* (*S. aureus*), *Haemophilus influenza*, a beta-group *Streptococcus*, *Yersinia enterocolitica*, *Klebsiella pneumonia*, F’s dysentery bacillus, *Salmonella typhi* and *Pseudomonas aeruginosa*, with MICs of 172.90, 172.90, 172.90, 172.90, 172.90, 86.45, 172.90, 345.80, 518.70 and 864.50 μg/mL, respectively [112,113]. The mechanism might be closely related to the disruption of the cell membrane and degradation of bacterial proteins [112]. Ethanol, methanol and aqueous extracts of *Forsythiae Fructus* also exhibited antibacterial activity [114,115,116]. Li et al. [114] found that the ethanol extract remarkably decreased secretion of α-hemolysin in *S. aureus* at a concentration of 16–128 mg/L. Han et al. [115] demonstrated that the aqueous extract inhibited growth of *E. coli*, *S. aureus* and *Salmonella* in a dose-dependent manner, indicating its uses in broiler chickens as a substitute antibiotic in vivo. The active compounds of *Forsythiae Fructus* were assessed for their antibacterial activities by *E. coli*, *Pseudomonas aeruginosa*, *S. aureus*, *Helicobacter pylori* and *Klebsiella pneumoniae*. As a result, the MIC values of forsythoside A (**1**), isoforsythoside A (**30**), phillyrin (**60**), 3β-hydroxyanticopalic acid (**124**), agatholic acid (**125**), β-amyrin acetate (**155**), 3β-acetoxy-20α-hydroxyursan-28-oic acid (**159**), betulinic acid (**160**) and ψ-taraxasterol (**173**) for *E. coli* were 38.33, 40.83, 3.94, 3.42, 2.62, 5.00, 4.55, 1.20 and 1.20 μg/mL, respectively [27,48]. The MIC values of forsythoside A (**1**) and isoforsythoside A (**30**) were 38.33 and 40.83 μg/mL, respectively, for *Pseudomonas aeruginosa* and 76.67 and 81.66 μg/mL for *S. aureus* [27]. In addition, some studies indicated that the antibacterial effect of *Forsythiae Fructus* was related to its inhibitory effect on the efflux pump of bacteria, but these studies are still in a primary stage [116].

### 4.3. Antiviral Effect

The antiviral effect of *Forsythia Fructus* mainly focused on influenza A (H1N1) virus, respiratory syncytial virus (RSV) and infectious bronchitis virus (IBV). Previous studies suggested that the 80% ethanol extract of *Forsythia Fructus* protected H1N1-infected MDCK cells with a minimal inhibitory concentration (MIC) of 1:8192 mg/mL [8]. Ko et al. [117] found that the 95% ethanol, 50% ethanol and aqueous extracts exhibited a dual regulatory effect on H1N1-infected human bronchial epithelial cells with IC_50_ values of 42 ± 6, 117 ± 15 and 232 ± 28 g/mL, respectively. Four compounds from *Forsythia Fructus*, namely forsythoside A (**1**), calceolarioside B (**33**), phillyrin (**60**) and rengynic acid (**108**), also demonstrated significant antiviral activity. In vivo, forsythoside A (**1**) at 20 ug/kg was able to control H1N1 infection and relieved the symptoms by inhibiting expression of Toll-like receptor 7 (TLR7), MyD88, tumor necrosis factor receptor-associated factor 6 (TRAF6), interleukin-4 receptor-associated kinase (IRAK4) and NF-kB p65 mRNA and H1N1 replication in C57BL/6j mice [77]. Phillyrin (**60**) inhibited H1N1 expresstion by down-regulating the gene of the H1N1 nucleoprotein in vitro and in vivo [118,119]. Meanwhile, forsythoside A (**1**), calceolarioside B (**33**) and rengynic acid (**108**) exhibited good anti-RSV effects in multiple different cell lines [23,43,120]. In addition, forsythoside A (**1**) was able to inhibit IBV in primary chicken embryo kidney cells at a concentration of 0.16 to 0.64 mm and in HD11 cells at a concentration from 10 uM/L to 20 uM/L, suggesting its potential for preventing IBV infection [121,122].

### 4.4. Antioxidant Effect

Recently, some studies revealed the anti-oxidative effect of the *Forsythia Fructus* extract and its compounds in the 2,2-diphenyl-1-picrylhydrazyl (DPPH), 2,2′-azino-bis-3-ethylbenzothiazoline-6-sulfonic acid (ABTS) and ferric reducing antioxidant power (FRAP) assays in vitro [25,27,28,123,124,125]. The results indicated that forsythoside A (**1**), isoforsythoside A (**30**), phillygenin (**59**), phillyrin (**60**), forsythialan A (**88**) and polysaccharides exhibited strong antioxidant effects, with the DPPH IC_50_ values of 0.43, 2.74, 53.64, 351.14, 29.86 μg/mL and 0.08 mg/mL, respectively [27,123,124]. Calceolarioside C (**21**), forsythoside H (**38**) and lianqiaoxinoside B (**43**) were tested by the ABTS test and exhibited IC_50_ values of 22.7, 17.7 and 15.6 μg/mL, respectively [25,28]. Additionally, the ethyl acetate extract of *Forsythia Fructus* showed a strong antioxidant activity by the DPPH and FRAP assays [125]. Phillygenin (**59**) and 8-hydroxypinoresinol (**73**) at 50 μM were confirmed to reverse a LLC-PK1 cell damage induced by 3-morpholinosydnonimine, an ONOO-generator [126]. In addition, eight lignans—phillygenin (**59**), 7′-epi-8-hydroxypinoresinol (**63**), pinoresinol (**68**), 8-hydroxypinoresinol (**73**), isolaraciresinol (**78**), cedrusin (**82**), olivil (**94**) and lariciresinol (**98**) exerted inhibitory effects against lipid peroxidation of high-density lipoprotein (HDL) induced by AAPH (a thermo-labile radical generator), with IC_50_ values ranging from 12.1 to 51.1 μM [32]. In vivo, pretreatment with a CH_2_Cl_2_ fraction of *Forsythia Fructus* 80% ethanol extract inhibited oxidative stress in diquat-treated rats. The mechanism was associated with an increase in the activities of superoxide dismutase (SOD) and glutathione peroxidase (GSH-Px) as well as the levels of GSH in plasma, liver and kidney, whereas a reduction in the level of malondialdehyde (MDA) was observed in plasma and the kidney [124]. Yan et al. [127] found that the anti-aging effect of phillyrin (**60**) is closely related to the antioxidant effect in aging model mice. Interestingly, the *Forsythia Fructus* extract has been used as an animal feed additive in weaned piglets and Arbor Acre broilers, mainly due to the improvement in growth performance via the modulation of some endogenous antioxidant molecules and oxidative stress biomarkers (SOD, GSH-Px and MDA) [128,129,130].

### 4.5. Neuroprotective Effect

The neuroprotective effect is a newly established research direction for *Forsythiae Fructus*. Zhang et al. [131] found that the *Forsythiae Fructus* ethanol extract reduced rotenone toxicity and protected PC12 cells. Further in vivo study demonstrated that *Forsythiae Fructus* (50 and 200 mg/kg) exhibited a protective effect in rotenone-stimulated rats through down-regulating inflammatory and oxidation factors. Forsythoside A (**1**) was the main compound with neuroprotective effects reported in *Forsythiae Fructus*. It ameliorated the physiology of senescence-accelerated mouse prone (SAMP8) mice and scopolamine-induced memory deficit mice, with significant increase in total superoxide dismutase (T-SOD), choline acetyl transferase (ChAT) and GSH-Px activities; significant decrease in MDA and NO levels; inhibition of AchE activity and increase of p-ERK expression, indicating that its mechanism might be to regulate the cholinergic system and antioxygenation [132,133,134]. Furthermore, cognitive functions of gerbils with transient cerebral global ischemia were ameliorated after treatment with forsythoside A (**1**) at 10 mg/kg due to the inhibition of activated microglia and astrocytes [135]. In vitro, forsythoside A (**1**) significantly inhibited the cell apoptosis induced by Aβ_25-35_ in PC12 and HT22 cells, which are closely related to Alzheimer’s disease [136,137]. Moreover, phillyrin (**60**) protected SH-SY5Y neuroblastoma cells against MPP^+^ [138], while forsythoneoside B (**191**) and forsythoneoside D (**193**) at 0.1 μM significantly inhibited PC12 cell damage induced by rotenone and increased cell viability [11], indicating their potential toward Parkinson’s disease.

### 4.6. Antitumor Effect

*Forsythiae Fructus* aqueous extract treatment of B16-F10 melanoma-transplanted C57BL/6 mice inhibited cancer cell proliferation and angiogenesis and prolonged their survival time, indicating a noticeable antitumor activity. The results revealed that this effect has a close relationship with antioxidant and anti-inflammatory activities via the MAPKs/Nrf2/HO-1 pathway [7]. The LQ-4 extract (which contains at least twelve types of compounds) showed antitumor actions on Hela and SGC-7901 cells by inhibiting cell proliferation and inducing apoptosis, which were probably related to the decomposition of caspase-8 protease [139,140,141]. Phillyrin (**60**) exhibited an antitumor effect on Lewis lung carcinoma in vivo at three doses of 5, 10 and 20 g/kg/d by decreasing vascular endothelial growth factor (VEGF) expression and increasing endostatin expression [142]. In addition, (+)-8-hydroxyepipinoresinol-4-*O*-β-d-glucopyranoside (**65**) showed significant cytotoxicity to A549, Colo205, Hep-3B, HL60 and KB cancer cell lines with IC_50_ values of 9.48, 7.75, 0.59, 4.06 and 38.38 μM, respectively [34]. Moreover, dammar-24-en-3β-acetoxy-20-ol (**147**) and ambrolic acid (**163**) from *Forsythiae Fructus* were tested against SGC-7901 and PC-3 cells. Both of them induced apoptosis of SGC-7901 cells dose-dependently by down-regulating the expression of caspase proteins (caspase 3, 6, 8 and 9) and up-regulating the levels of Bax [51,55], whereas, dammar-24-en-3β-acetoxy-20-ol (**147**) might also inhibit the activities of telomerases in PC-3 cells, thus enhancing the radiosensitivity of PC-3 cells [143].

### 4.7. Hepatoprotective Effect

The active compound phillygenin (**59**) in *Forsythiae Fructus* has been shown to exhibit a protective effect against acute liver injury induced by CCl_4_ in rats at the dosages of 0.05, 0.15, 0.5 mg/kg. It increased the activities of SOD, GSH-Px and GSH; decreased MDA and reduced the levels of TNF-α and IL-8 in liver tissue [10]. Wang et al. [144] reported that Lian qiao gan yuan (phillygenin) protected against hepatic fibrosis induced by bovine serum albumin in rats. However, the author considered forsythoside A (**1**) to be Lian qiao gan yuan in Chinese. Forsythin (**60**) showed a protective capability against alcohol-induced liver injury by suppressing expression of apoptosis factors (PARP and caspase 3) [145]. Moreover, the aqueous extract of *Forsythiae Fructus* excerted a hepatoprotective effect in liver injured rats with acute pancreatitis at three dosages of 1.25, 2.5 and 5.0 g/kg. This was associated with inhibition of mRNA expression of NF-κB and Foxp3, subsequently reducing activation of the NF-κB signaling pathway, which plays an important role in the pathogenesis of severe acute pancreatitis [146].

### 4.8. Cardiovascular Protective Effect

The cardiovascular protective activity of *Forsythiae Fructus* and its compounds has been reported in recent years. In an in vivo study, oral administration of ethyl acetate extract at dosages of 50, 100 and 200 mg/kg improved pathological damage and increased the serum level of insulin as well as expression of pancreatic function genes (PDX-1, INS-1 and INS-2) in streptozotocin-induced diabetic mice, indicating its potency as an antihyperglycemic and antihyperlipidemic agent [147]. Treatment with 150 mg/kg phillyrin (**60**) for ten weeks in an atherosclerosis (AS) model noticeably reduced the area of AS plaques, improved the function of arterial condensation and inhibited expression of sodium hydrogen exchange protein 1 (NHE-1), intercellular cell adhesion molecule-1 (ICAM-1), vascular cell adhesion molecule-1 (VACM-1), IL-1 and IL-6 [12]. Moreover, forsythoside A (**1**) exhibited a vasorelaxant effect against norepinephrine-stimulated vasocontraction in rats by decreasing calcium influx from the extracellular space [148]**.**

### 4.9. Others

The aqueous extract of *Forsythiae Fructus* reduced the serum gastrin content and promoted gastrointestinal movement, demonstrating an anti-vomiting effect in mice exposed to chemotherapy [9]. Phillyrin (**60**) was shown to exert a remarkable antiobesity effect in high glucose-induced lipid accumulation in HepG2 cells and 3T3-L1 adipocytes, as well as in obese mice [149,150,151]. The mechanism of action was possibly due to inducing the liver kinase B1 (LKB1) phosphorylation and activating AMP-activated protein kinase (AMPK), thus reducing expression of sterol regulatory element-binding protein-1c (SREBP-1c) and fatty acid synthase. Interestingly, forsythoside A (**1**) exhibited antiandrogenic alopecia activity in dihydrotestosterone-stimulated mice by suppressing the apoptosis of hair cells [152]. Forsythoside A (**1**) also exhibited an immune regulation effect in endotoxemia mice by down-regulating mRNA expression of Foxp3 and decreasing the secretion of IL-10 and TNF-α [153]. Moreover, in yeast-stimulated pyrexia mice, forsythoside A (**1**) increased the expression of temperature-sensitive transient receptor potential A1 (TRPA1), thereby taking antipyretic effect [154]. Furthermore, a study demonstrated that forsythoside A inhibited P-gp ATPase activity, thus influencing the efflux of drugs [155].

## 5. Pharmacokinetics

Pharmacokinetic studies have provided a scientific basis for the clinical application of *Forsythiae Fructus* and the data were presented in Table 4. When Sprague Dawley (SD) rats were orally administrated UFF and RFF extract, the main active compounds of forsythoside A (**1**), phillyrin (**60**), rutin (**175**), quercetin (**177**) and isorhamnetin (**180**) showed very different pharmacokinetic parameters, including *C*_max_, *AUC*_0–24 h_ and *T*_max_. Generally, the *AUC*_0–24 h_ and *C*_max_ were much higher in the UFF group than in the RFF group. The absorption was faster after oral administration of UFF, as reflected by *T*_max_, whereas quercetin (**177**) and isorhamnetin (**180**) couldn’t be detected after RFF treatment. The pharmacokinetic properties after multiple-dose treatment had significantly increased than those after single-dose treatment, indicating that the harvest times affected the contents and bioavailability of active compounds in *Forsythiae Fructus* [59]. Liu et al. [31] developed an HPLC-ESI-MS/MS method for the quantification of matairesinol-4′-*O*-glucoside (**58**), phillygenin (**59**), phillyrin (**60**), (+)-pinoresinol-β-d-glucoside (**69**) and hyperin (**182**) in rat bile after oral administration of 75% methanol extract of *Forsythiae Fructus*, revealing that bile was the major pathway for the excretion of lignans in *Forsythiae Fructus*. Forsythoside A (**1**), phillygenin (**59**) and phillyrin (**60**) were the three most studied compounds in *Forsythiae Fructus* for pharmacokinetics. After oral administration of forsythoside A, the absorption was fast with a *T*_max_ of 20 min, but the bioavailability was only 0.5% [158]. Furthermore, Chen et al. [159] revealed that most of forsythoside A (**1**) was excreted through bile due to the bile-to-blood distribution ratio was 0.32 ± 0.06 after intravenous administration. Phillyrin (**60**) was absorbed into plasma through passive diffusion and could be influenced by P-gp, thus exhibiting a low bioavailability [160]. After oral administration, a total of thirty-four metabolites of phillyrin (**60**) were found in rat bile, urine and feces by UPLC-Q-TOF-MS, providing a basis for the pharmacological activities of phillyrin in vivo, and the results also revealed that deglucosidation was the main metabolic reaction for phillyrin [161]. Absorption of phillygenin (**59**) was linear at three dosages of 1.4, 2.8, and 5.6 mg/kg, but it showed a rapid elimination rate of approximately 6 min [162]. In addition, forsythoside A induced the activities of CYP1A2 and CYP2C11, while phillyrin induced the activities of CYP1A2 and CYP2D1, which provided very useful information about interactions in the combination drug therapy [163].

## 6. Conclusions

In Asia, *Forsythiae Fructus* is widely used in the clinic as a single drug or compound prescription. Modern pharmacology showed that it has a variety of bioactivities, including anti-inflammatory, antibacterial, antiviral, antioxidant, antitumor, antidiabetic, antihyperlipidemic, antiandrogenic alopecia, antivomiting, antiaging and anti-obesity activities, as well as neuroprotective, hepatoprotective and vasorelaxant effects. In the past few years, many sensitive analysis technologies have been used for research of this herb. Three hundred and twenty-one compounds have been identified, including fifty-one phenylethanoid glycosides, fifty lignans, nineteen aliphatic alcohols with the C6-C2 skeleton, two iridoids, nineteen diterpenoids, twenty-seven triterpenoids, six sterols, nineteen flavonoids, fifty-two volatiles, seven alkaloids, twenty-eight organic acids, six amino acids, nine sugar derivatives, two allylbenzene glycosides and twenty-four others. Among them, forty-five were from the UFF, twenty-two were from the RFF, twenty-one were from the UFF and RFF and the remaining compounds have not been mentioned from UFF or RFF.

Moreover, phenylethanoid glycosides (forsythoside A), lignans (phillyrin, arctiin) and flavonoids (rutin, forsythoneoside D) are the major constituents and exerted various bioactivities, such as anti-inflammatory, antiviral, and neuroprotective effects. Additionally, the different harvest times not only affected the contents but also the bioavailabilities of the active compounds, especially forsythoside A and phillyrin. However, few studies have reported the difference in pharmacological activities between UFF and RFF. Altogether, this review extensively summarized the phytochemistry, quality control, pharmacology and pharmacokinetics of *Forsythiae Fructus*, especially the UFF and RFF, and provided evidence for its further research and clinical applications.

## Figures and Tables

**Figure 1:** Chemical structures of phenylethanoid glycosides in *Forsythiae Fructus*.

**Table:** 

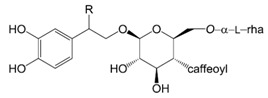	**Compounds**	**R**
	Forsythoside A (**1**)	H
	Forsythoside C (Suspensaside, **2**)	OH
	(*R*)-Suspensaside (**3**)	β-OH
	(*S*)-Suspensaside (**4**)	α-OH
	(*S*)-Suspensaside methyl ether (**5**)	α-OCH_3_
	Suspensaside B (**6**)	OC_4_H_9_
	(*R*)-Forsythoside J (**7**)	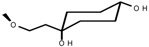
	(*S*)-Forsythoside J (**8**)	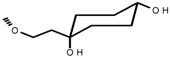
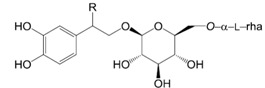	**Compounds**	**R**
	Forsythoside D (**9**)	OH
	Forsythoside E (**10**)	H
	β-Methoxyforsythoside E (**11**)	OCH_3_

**Table:** 

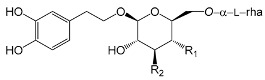	**Compounds**	**R_1_**	**R_2_**
	Iso-forsythoside A/Forsythoside I/Lianqiaoxinside A (**12**)	OH	caffeoyl
	ForsythosideA-4’*O*-β-d-glucopyranoside (**13**)	(4’*O*-β-d-glu) caffeoyl	OH
	Forsythenside K (**14**)	coumaroyl	OH
	Poliumoside (**15**)	caffeoyl	*O*-β-l-rha

**Table:** 

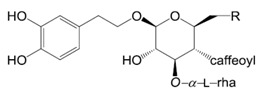	**Compounds**	**R**
	Acteoside (**16**)	OH
	Forsythoside B (**17**)	*O*-api
	Forsythoside G (**18**)	2-*O*-methyl-api
	Forsythoside F (**19**)	*O*-β-d-xyl
	Angoroside A (**20**)	*O*-arabinose
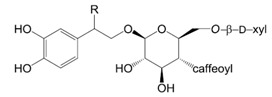	**Compounds**	**R**
	Calceolarioside C (**21**)	H
	(*S*)-β-hydroxycalceolarioside C (**22**)	α-OH
	(*R*)-β-hydroxycalceolarioside C (**23**)	β-OH
	(*S*)-β-methoxycalceolarioside C (**24**)	α-OCH_3_
	(*R*)-β-methoxycalceolarioside C (**25**)	β-OCH_3_

**Table:** 

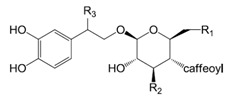	**Compounds**	**R_1_**	**R_2_**	**R_3_**
	Calceolarioside A (**26**)	OH	OH	H
	Derhamnosyl suspensaside (**27**)	OH	OH	OH
	β-methoxylacteoside (**28**)	OH	*O*-α-l-rha	OCH_3_
	Caffeoyl calceolarioside C (**29**)	*O*-β-d-glc	*O*-api	H
	Isoforsythiaside (**30**)	*O*-β-l-rha	OH	H

**Table:** 

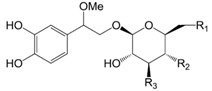	**Compounds**	**R_1_**	**R_2_**	**R_3_**
	β-Methoxyferruginoside B (**31**)	*O*-β-d-glc	OH	OH
	β-Methoxylipedoside A (**32**)	OH	coumaroyl	*O*-α-l-rha

**Table:** 

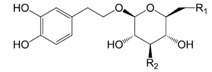	**Compounds**	**R_1_**	**R_2_**
	Calceolarioside B (**33**)	caffeoyl	OH
	Lianqiaoxinoside C (**34**)	*O*-β-d-xyl	caffeoyl
	Plantainoside A (**35**)	OH	caffeoyl

**Table:** 

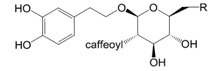	**Compounds**	**R**
	Forsythoside J (**36**)	*O*-β-d-xyl
	Plantainoside B (**37**)	OH
	Forsythoside H (**38**)	*O*-α-l-rha

**Table:** 

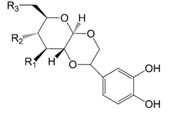	**Compounds**	**R_1_**	**R_2_**	**R_3_**
	Suspensaside A (**39**)	OH	caffeoyl	*O*-α-l-rha
	Suspensaside A isomer (**40**)	OH	caffeoyl	coumaroyl
	Demethylsuspensaside A (**41**)	OH	caffeoyl	*O*-xyl/*O*-api
	Suspensaside C (**42**)	OH	OH	*O*-α-l-rha
	Lianqiaoxinoside B (**43**)	caffeoyl	OH	*O*-α-l-rha

**Table:** 

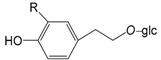	Salidroside R = H (**44**)
	3,4-Dihydroxyphenylethyl-8-*O*-β-d-glucopyranoside R = OH (**45**)
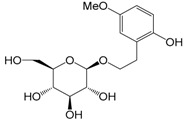	Forsythiayanoside C (**46**)
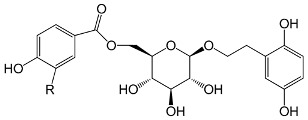	2-(2,5-Dihydroxyphenyl)-ethyl-*O*-(6-*O*-*p*-hydroxybenzoyl)-d-glucopyranoside R = H (**47**)
	2-(2,5-Dihydroxyphenyl)-ethyl-*O*-(6-ovanilloyl)-d-glucopyranoside R = OCH_3_ (**48**)
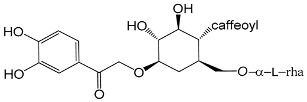	2-(3,4-Dihydroxyphenyl)-2-oxo-ethyl-*O*-l-rhamnopyranosyl-(16)-(4-*O*-caffeoyl)-d-glucopyranoside. (**49**)
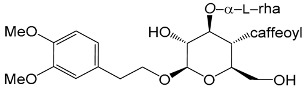	Brachynoside (**50**)
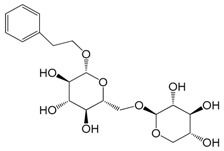	Phenethyl alcohol β-d-xylopyranosyl-(1→6)-β-d-glucopyranoside (**51**)

**Figure 2:** Chemical structures of lignans in *Forsythiae Fructus*.

**Table:** 

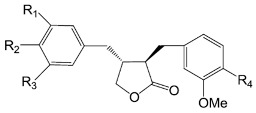	**Compounds**	**R_1_**	**R_2_**	**R_3_**	**R_4_**
	Arctigenin (**52**)	OCH_3_	OCH_3_	H	OH
	Arctiin (**53**)	OCH_3_	OCH_3_	H	*O*-glu
	Matairesinoside (**54**)	OCH_3_	OH	H	*O*-glu
	Matairesinol (**55**)	OCH_3_	OH	H	OH
	2′,5′-Dihydroxy-4′′-caffeoyl matairesinol (**56**)	OCH_3_	OH	OH	caffeoyl
	3′,4′,5′-Trihydroxy-3′′-methoxy-4′′-caffeoyl lignan (**57**)	OH	OH	OH	caffeoyl
	Matairesinol-4′-*O*-glucoside (**58**)	OCH_3_	*O*-β-d-glc	H	OH

**Table:** 

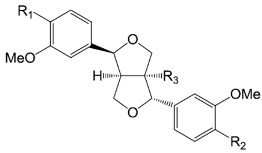	**Compounds**	**R_1_**	**R_2_**	**R_3_**
	Phillygenin (**59**)	OCH_3_	OH	H
	Phillyrin (forsythin **60**)	OCH_3_	*O*-β-d-glc	H
	Caffeoyl phillygenin (**61**)	OCH_3_	caffeoyl	H
	(+)-Epipinoresinol (**62**)	OH	OH	H
	7′-Epi-8-hydroxypinoresinol (**63**)	OH	OH	OH
	(+) Epipinoresinol-4-β-d-glucoside (**64**)	OH	*O*-β-d-glc	H
	(+)-8-Hydroxyepipinoresinol-4-*O*-β-d-glucopyranoside (**65**)	OH	*O*-β-d-glc	OH
	(+) epipinoresinol-4′-β-d-glucoside (**66**)	*O*-β-d-glc	OH	H
	forsythialanside E (**67**)	*O*-β-d-glc	OH	OH
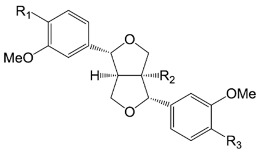	**Compounds**	**R_1_**	**R_2_**	**R_3_**
	(+) Pinoresinol (**68**)	OH	H	OH
	(+) Pinoresinol-β-d-glucoside (**69**)	*O*-β-d-glc	H	OH
	(+) Pinoresinol monomethyl ether-β-d-glucoside (**70**)	*O*-β-d-glc	H	OCH_3_
	Pinoresinol diglucoside (**71**)	*O*-β-d-glc	H	*O*-β-d-glc
	Caffeoyl pinoresinol (**72**)	caffeoyl	H	OH
	(+)-1-Hydroxypinordsinol (**73**)	OH	OH	OH
	(+)-1-Hydroxypinordsinol-4′-*O*-β-d-glucoside (**74**)	OH	OH	*O*-β-d-glc
	(+)-1-Hydroxypinordsinol-4′′-*O*-β-d-glucoside (**75**)	*O*-β-d-glc	OH	OH
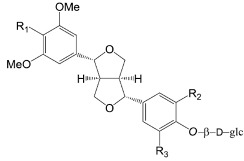	**Compounds**	**R_1_**	**R_2_**	**R_3_**
	3′,4′,5′-Trimethoxyl-4′′-hydroxyllignan *O*-glucoside (**76**)	OCH_3_	H	H
	Syringaresinol-4-*O*-β-d-glucoside (**77**)	OH	OCH_3_	OCH_3_

**Table:** 

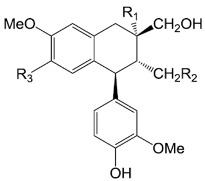	**Compounds**	**R_1_**	**R_2_**	**R_3_**
	Isolariciresinol (**78**)	H	H	H
	Isolariciresinol-4-*O*-β-d-glucopyranoside (**79**)	H	H	*O*-β-d-glc
	Isolariciresinol-9′-*O*-β-d-glucopyranoside (**80**)	H	*O*-β-d-glc	H
	Isoolivil (**81**)	OH	H	H
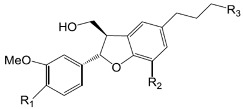	**Compounds**	**R_1_**	**R_2_**	**R_3_**
	Cedrusin (**82**)	OH	OH	OH
	Glochidioboside (**83**)	OH	OCH_3_	*O*-glc
	Forsythialanside C (**84**)	*O*-glc	OCH_3_	*O*-rha
	Forsythialanside D (**85**)	*O*-rha	OCH_3_	*O*-rha
	Dihydrodehydrodiconiferyl alcohol-4-*O*-β-d-glucoside (**86**)	*O*-glc	OCH_3_	OH

**Table:** 

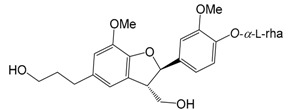	Icariside E_4_ (**87**)
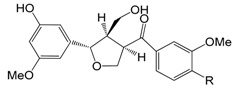	Forsythialan A R = OH (**88**)
	Forsythialan B R = OMe (**89**)
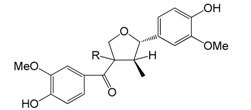	*rel*-(7*R*,8′*R*,8*S*)-Forsythialan C R = β-H (**90**)
	*rel*-(7*R*,8′*R*,8*R*)-Forsythialan C R = α-H (**91**)
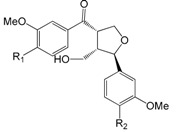	Forsythialanside A R_1_ = OMe R_2_ = *O*-glc (**92**)
	Forsythialanside B R_1_ = *O*-glc R_2_ = OH (**93**)
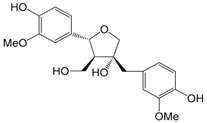	Olivil (**94**)
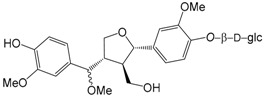	Forsythiayanoside B (**95**)
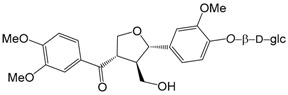	Forsythiayanoside A (**96**)
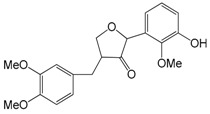	3-Furanone-2-(3-methoxy-4-hydroxyphenyl)-4-veratryl (**97**)
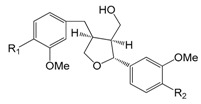	Lariciresinol R_1_ = OH R_2_ = OH (**98**)
	Lariciresinol-4-*O*-β-d-glucoside R_1_ = OH R_2_ = *O*-β-d-glc (**99**)
	Lariciresinol-4′-*O*-β-d-glucoside R_1_ = *O*-β-d-glc R_2_ = OH (**100**)
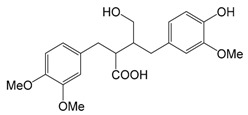	Benzenebutanoic acid (**101**)

**Figure 3:** Chemical structures of natural alcohols with the C_6_-C_2_ skeleton in *Forsythiae Fructus*.

**Table:** 

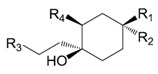	**Compounds**	**R_1_**	**R_2_**	**R_3_**	**R_4_**
	Isorengyol (**102**)	H	OH	OH	H
	Rengyol (**103**)	OH	H	OH	H
	Suspenol (**104**)	OH	H	OH	OH
	Rengyolester (**105**)	OH	H	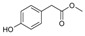	OH
	Rengyoside A (**106**)	OH	H	*O*-β-d-glc	H
	Rengyoside C (**107**)	OH	H	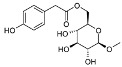	H

**Table:** 

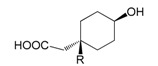	Rengynic acid R = OH (**108**)
	Rengynic acid-1′-*O*-β-d-glucopyranoside R = *O*-β-d-glc (**109**)
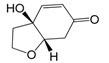	Rengyolone (halleridone) (**110**)
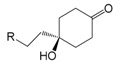	Rengyoxide R = OH (**111**)
	Rengyoside B R = *O*-β-d-glc (**112**)
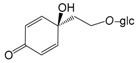	Cornoside (**113**)
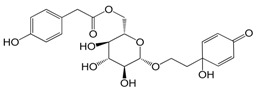	Forsythenside A (**114**)
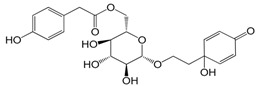	Forsythenside B (**115**)
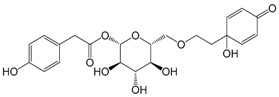	Forsythenside F (**116**)
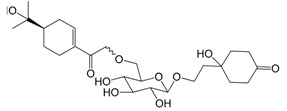	Forsythenside H (**117**)
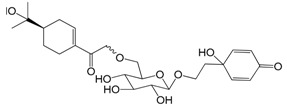	Forsythenside G (**118**)
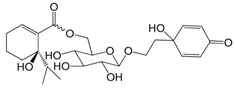	Forsythenside I (**119**)
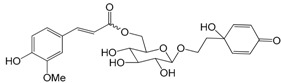	Forsythenside J (**120**)

**Figure 4:** Chemical structures of iridoids, diterpenoids, terpenoids in *Forsythiae Fructus*.

**Table:** 

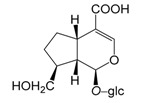	Adoxosidic acid (**121**)
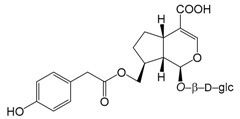	Adoxosidic acid 10-*p*-hydroxyphenylacetate (**122**)
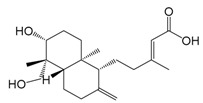	3β-Hydroxylabda-8(17), 13*E*-dien-15-oic acid (**123**)
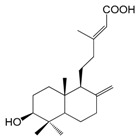	3β-Hydroxyanticopalic acid (**124**)
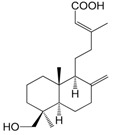	Agatholic acid (**125**)
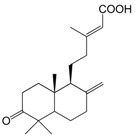	3-Oxoanticopalic acid (**126**)

**Table:** 

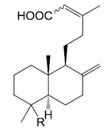	**Compounds**	**R**
	19-Hydroxylabda-8(17),13(*Z*)-dien-15-oic acid (**127**)	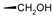
	19-Hydroxylabda-8(17),13(*E*)dien-15-oic acid (**128**)	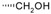
	19-Formyllabda-8(17),13(*E*)-dien-15-oic acid (**129**)	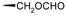
	19-Formyllabda-8(17),13(*Z*)-dien-15-oic acid (**130**)	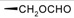
	Labda-8(17),13(*Z*)-dien-15,18-dioic acid (**131**)	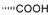

**Table:** 

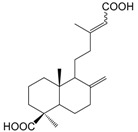	Labda-8(17),13(*Z*)-diene-15,19dioic acid (**132**)
	Labda-8(17),13(*E*)-diene-15,19-dioic acid (**133**)
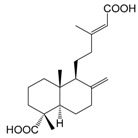	Dehydropinifolic acid (**134**)
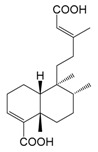	Haplopappic acid (**135**)
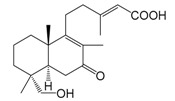	18-Hydroxy-7-oxolabda-8(9),13(*E*)-dien-15-oic acid (**136**)
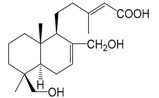	19-Dihydroxylabda-7(8),13(*E*)-dien-15-oic acid (**137**)
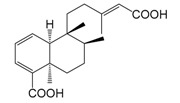	Forsythidin A (**138**)
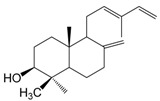	3β-Hydroxy-12,13(*E*)-biformene (**139**)
	3β-Hydroxy-12,13(*Z*)-biformene (**140**)
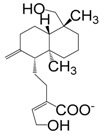	19-Hydroxy-8(17)(*E*)-13-labdadien-15-oate (**141**)
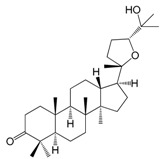	Ocotillone (**142**)
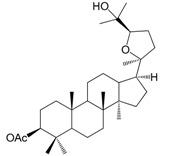	Ocotillol acetate (**143**)
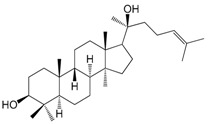	Garcinielliptone Q (**144**)
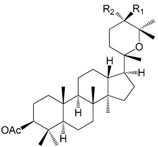	3β-Acetyl-20,25-epoxydammarane-24α-ol R_1_ = H,R_2_ = OH (**145**)
	3β-Acetyl-20,25-epoxydammarane-24β-ol R_1_ = OH,R_2_ = H (**146**)
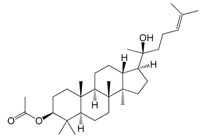	Dammar-24-en-3β-acetoxy-20-ol (**147**)
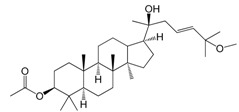	3β-Acetoxy-25-methoxydammar-23-en-20β-ol (**148**)
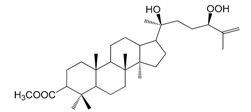	3β-Acetoxy-20S,24R-dammarane-25-ene-24-hydroperoxy-20-ol (**149**)
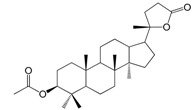	Cabralea lactone 3-acetate (**150**)
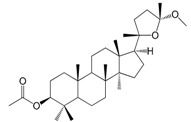	Cabralea lactone 3-acetate 24-methyl ether (**151**)
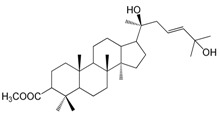	3-Acetylisofouquierol (**152**)

**Table:** 

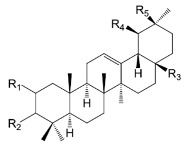	**Compounds**	**R_1_**	**R_2_**	**R_3_**	**R_4_**	**R_5_**
	Oleanolic acid (**153**)	H	β-OH	COOH	H	Me
	3β-Acetyloleanolic acid (**154**)	H	β-OAc	COOH	H	Me
	β-Amyrin acetate (**155**)	H	β-OAc	Me	H	Me
	Ursolic acid (**156**)	H	β-OH	COOH	Me	H
	2α,3α-Hydroxyursolic acid (**157**)	α-OH	α-OH	COOH	Me	H

**Table:** 

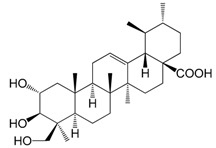	2α,23-Hydroxyursolic acid (**158**)
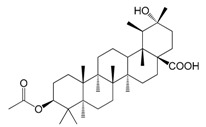	3β-Acetoxy-20α-hydroxyursan-28-oic acid (**159**)
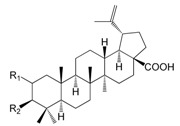	Betulinic acid R_1_ = H R_2_ = OH (**160**)
	3β-Acetylbetulinic acid R_1_ = H R_2_ = OAc (**161**)
	2α-Hydroxybetulinic acid R_1_ = α-OH R_2_ = OH (**162**)
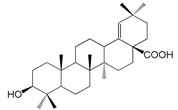	Ambrolic acid (**163**)
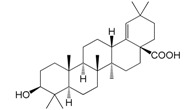	Morolic acid (**164**)
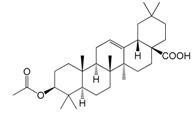	3β-Acetoxyolean-12-en-28-oic acid (**165**)
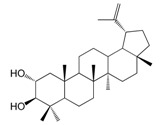	Alphitolic acid (**166**)
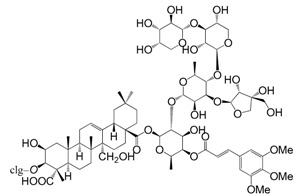	Onjisaponin F (**167**)
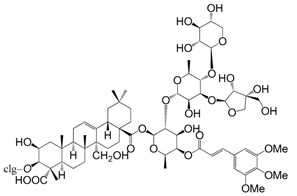	Onjisaponin G (**168**)

**Figure 5:** Chemical structures of sterols in *Forsythiae Fructus.*

**Table:** 

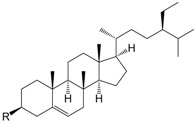	β-Sitosterol R = OH (**169**)
	Daueosterol R = *O*-β-d-glc (**170**)
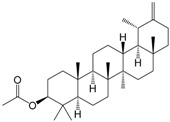	Taraxasterol acetate (**171**)
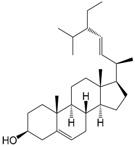	Stigmasterol (**172**)
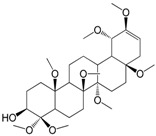	ψ-Taraxasterol (**173**)
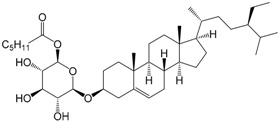	(6′-*O*-Palmitoyl)-sitosterol-3-*O*-β-d-glucoside (**174**)

**Table:** 

**Compounds**	**R_1_**	**R_2_**	**R_3_**	**R_4_**	**R_5_**
Rutin (**175**)	OH	OH	*O*-β-d-glc-*O*-α-l-rha	OH	H
Quercetin (**177**)	OH	OH	OH	OH	H
Isorhamnetin (**180**)	OCH3	OH	OH	OH	H
Kaempferfol (**181**)	OH	H	OH	OH	H
Hyperin (**182**)	OH	OH	*O*-β-d-gal	OH	H
Kaempferol-3-*O*-β-d-glucopyranoside-7-*O*-α-l-rhamnopyranoside (**185**)	OH	H	*O*-β-d-glc	*O*-α-l-rha	H
Kaempferol-3-*O*-β-d-(2″-*O*-β-d-glucopyranosyl-6″*O-*α-l-rhamnopyranosyl)glucopyranoside (**186**)	OH	H	*O*-β-d-(2″-*O*-β-d-glc-6″*O*-α-l-rha)glc	OH	H
Wogonin-7-*O*-glcoside (**187**)	H	H	H	*O*-β-d-glc	OMe
Baicalin (**188**)	H	H	H	*O*-glc	OH

**Table:** 

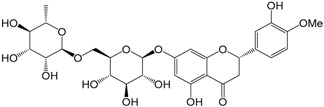	Hesperidin (**190**)
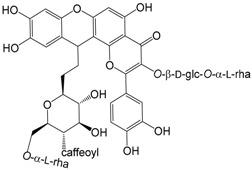	Forsythoneoside A 7′R (**191**)
	Forsythoneoside B 7′S (**192**)
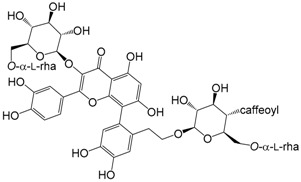	Forsythoneoside C M configuration (**193**)
	Forsythoneoside D P configuration (**194**)

**Figure 7:** Chemical structures of alkaloids in *Forsythiae Fructus.*

**Table:** 

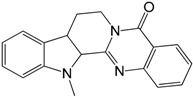	Rutaecarpine (**246**)
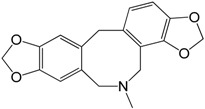	Suspensine A (**247**)
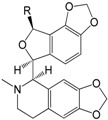	(−)-Egenine R = OH (**248**)
	(−)-7′-*O*-Methylegenine R = OMe (**249**)
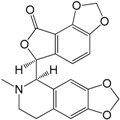	(−)-Bicuculline (**250**)
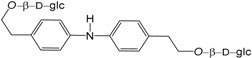	Bis-2-(4-aminophenyl) ethyl-β-d-glucopyranoside (**251**)
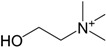	Choline (**252**)

**Figure 8:** Chemical structures of other compounds in *Forsythiae Fructus.*

**Table:** 

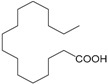	Palmitic acid (**253**)
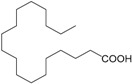	Stearic acid (**254**)
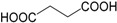	Succinic acid (**255**)
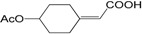	Suspenolic acid (**256**)
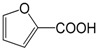	2-Furancarboxylic acid (**257**)
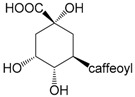	Chlorogenic acid (**258**)
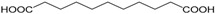	Anchoic acid (**259**)
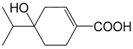	4-Hydroxy-4-isopropylcyclohex-1-enecarboxylic acid (**260**)
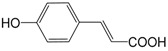	*p*-Coumaric acid (**261**)

**Table:** 

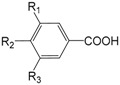	**Compounds**	**R_1_**	**R_2_**	**R_3_**
	Protocatechuic acid (**262**)	H	OH	OH
	Vanillic acid (**263**)	H	OH	OMe
	*p*-Hydroxybenzoic acid (**264**)	H	OH	H
	Benzoic acid (**265**)	H	H	H
	3,4-Dimethoxybenzoic acid (**266**)	H	OMe	OMe
	Syringic acid (**267**)	OMe	OH	OMe

**Table:** 

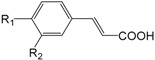	**Compounds**	**R_1_**	**R_2_**
	Caffeic acid (**268**)	OH	OH
	*trans*-Coumaric acid (**269**)	OH	H
	*trans*-Ferulic acid (**270**)	OH	OMe

**Table:** 

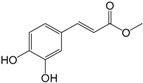	Caffeic acid methyl ester (**271**)
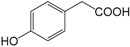	*p*-Hydroxybenzylacetic acid (**272**)
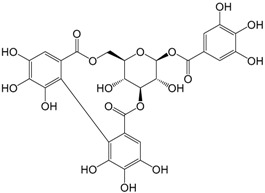	Tannic acid (**273**)
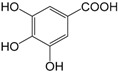	Gallic acid (**274**)
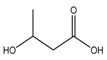	3-Hydroxybutyric acid (**275**)
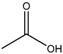	Acetic acid (**276**)
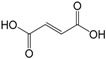	Pyruvic acid (**277**)
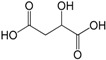	Malic acid (**278**)
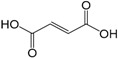	Fumaric acid (**279**)
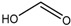	Formic acid (**280**)
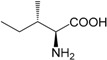	Isoleucine (**281**)
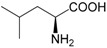	Leucine (**282**)
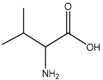	Valine (**283**)
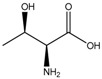	Threonine (**284**)
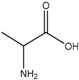	Alanine (**285**)
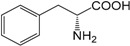	Phenylalanine (**286**)
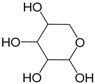	β-Xylose (**287**)
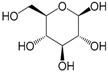	β-Glucose (**288**)
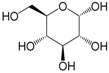	α-Glucose (**289**)
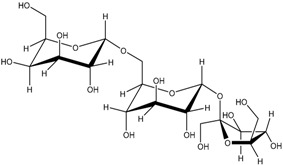	Raffinose (**290**)
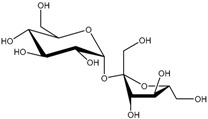	Sucrose (**291**)
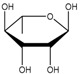	l-Rhamnose (**292**)
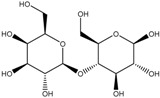	Lactose (**293**)
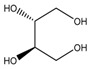	Erythritol (**294**)
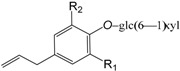	Forsythenside L R_1_ = H R_2_ = OH (**295**)
	Sasanquin R_1_ = OMe R_2_ = H (**296**)
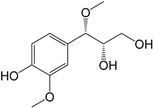	Forsythiayanoside D (**297**)
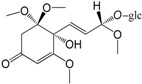	(6*S*,9*R*)-Roseoside (**298**)
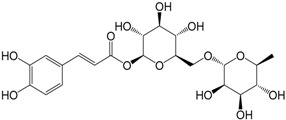	Swertiamacroside (**299**)
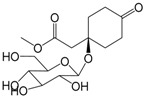	2,3,5,6-Tetrahydro-jacaranone-4-*O*-β-d-glucopyranoside (**300**)
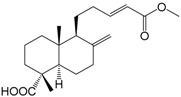	Labda-8(17),13E-dien-15,18-dioic acid 15-methyl ester (**301**)
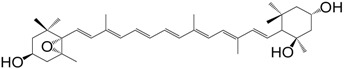	β-Carotene-5,6-epoxide (**302**)
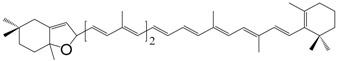	Mutatochrome (**303**)
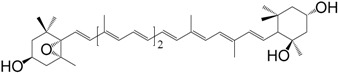	Neoxanthin (**304**)
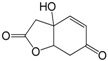	1-Oxo-4-hydroxy-2(3)-en-4ethylcyclohexa-5,8-olide (**305**)
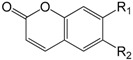	Esculetin R_1_ = OH,R_2_ = OH (**306**)
	6,7-Dimethoxycoumarin R_1_ = OMe, R_2_ = OMe (**307**)
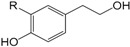	Hydroxytyrosol R = OH (**308**)
	*p*-Tyrosol R = H (**309**)
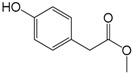	4-Hydroxybenylacetic acid methyl ester (**310**)
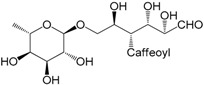	4-Caffeoylrutinose (**312**)
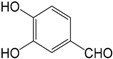	Protocatechualdehyde (**313**)
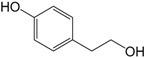	*p*-Hydroxyphenylethanol (**314**)
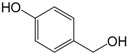	*p*-Hydroxybenzylalcohol (**315**)
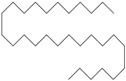	*n*-Hentriacontane (**316**)
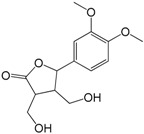	2,3-Dihydroxymethyl-4-(3′,4′-dimethoxyphenyl)-*γ*-butyrolactone (**317**)
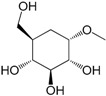	Methyl-α-d-glucopyranoside (**318**)
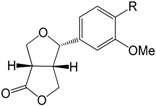	Forsythenin R = OMe (**319**)
	4-*O*-Demethylforsythenin R = OH (**320**)
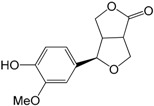	Salicifoliol (**321**)

**Table 1:** Compounds identified from *Forsythiae Fructus.*

NO.	Compound Name	Source		Reference
**Phenylethanoid Glycosides**				
**1**	forsythoside A (forsythiaside)	UFF, RFF	[6,14,15]	
**2**	forsythoside C (suspensaside)	RFF	[6,16]	
**3**	(*R*)-suspensaside	UFF	[17,18]	
**4**	(*S*)-suspensaside	UFF	[17,18]	
**5**	(*S*)-suspensaside methyl ether	N.M.	[18]	
**6**	suspensaside B	N.M.	[16]	
**7**	(*R*)-forsythoside J	N.M.	[19]	
**8**	(*S*)-forsythoside J	N.M.	[19]	
**9**	forsythoside D	N.M.	[20]	
**10**	forsythoside E	UFF	[20,21]	
**11**	β-methoxyforsythoside E	N.M.	[22]	
**12**	iso-forsythoside A/forsythoside I/lianqiaoxinside A	UFF	[15,17,21]	
**13**	forsythoside A 4′-*O*-β-d-glucopyranoside	N.M.	[11]	
**14**	forsythenside K (lipedoside A)	N.M.	[22,23]	
**15**	poliumoside	N.M.	[11]	
**16**	acteoside	N.M.	[22]	
**17**	forsythoside B	UFF	[17,22]	
**18**	forsythoside G	N.M.	[22]	
**19**	forsythoside F	UFF	[21,24]	
**20**	angoroside A	N.M.	[11]	
**21**	calceolarioside C	UFF	[25]	
**22**	(*S*)-β-hydroxycalceolarioside C	N.M.	[22]	
**23**	(*R*)-β-hydroxycalceolarioside C	N.M.	[22]	
**24**	(*S*)-β-methoxycalceolarioside C	N.M.	[22]	
**25**	(*R*)-β-methoxycalceolarioside C	N.M.	[22]	
**26**	calceolarioside A	N.M.	[26]	
**27**	derhamnosyl suspensaside	N.M.	[22]	
**28**	β-methoxyacteoside	N.M.	[22]	
**29**	caffeoyl calceolarioside C	N.M.	[22]	
**30**	isoforsythiaside	N.M.	[27]	
**31**	β-methoxylferruginoside B	N.M.	[22]	
**32**	β-methoxylipedoside A	N.M.	[22]	
**33**	calceolarioside B	UFF	[21]	
**34**	lianqiaoxinoside C	UFF	[25]	
**35**	plantainoside A	N.M.	[24]	
**36**	forsythoside J	UFF	[21]	
**37**	plantainoside B	N.M.	[24]	
**38**	forsythoside H	UFF	[21,24,28]	
**39**	suspensaside A	N.M.	[16,22]	
**40**	suspensaside A isomer	N.M.	[22]	
**41**	demethyl suspensaside A	N.M.	[22]	
**42**	suspensaside C	N.M.	[14]	
**43**	lianqiaoxinoside B	UFF	[28]	
**44**	salidroside	N.M.	[29]	
**45**	3,4-dihydroxyphenylethyl-8-*O*-β-d-glucopyranoside	UFF	[17]	
**46**	forsythiayanoside C	UFF	[30]	
**47**	2-(2,5-dihydroxyphenyl)-ethyl-*O*-(6-*O*-p-hydroxybenzoyl)-β-d-glucopyranoside	N.M.	[11]	
**48**	2-(2,5-dihydroxyphenyl)-ethyl-*O*-(6-*O*-vanilloyl)-β-d-glucopyranoside	N.M.	[11]	
**49**	2-(3,4-dihydroxyphenyl)-2-oxo-ethyl-*O*-α-l-rhamnopyranosyl-(1→6)-(4-*O*-caffeoyl)-β-d-glucopyranoside	N.M.	[11]	
**50**	brachynoside	N.M.	[22]	
**51**	phenethyl alcohol β-d-xylopyranosyl-(1→6)-β-d-glucopyranoside	UFF	[21]	
**Lignans**				
**52**	arctigenin	UFF	[17,22]	
**53**	arctiin	UFF	[17,22]	
**54**	matairesinoside	N.M.	[22]	
**55**	matairesinol	UFF	[17,22]	
**56**	2′,5′-dihydroxy-4′′-caffeoyl matairesinol	N.M.	[22]	
**57**	3′,4′,5′-trihydroxy-3′′-methoxyl-4′′-caffeoyl lignan	N.M.	[22]	
**58**	matairesinol-4′-*O*-glucoside	N.M.	[31]	
**59**	phillygenin	UFF, RFF	[15,32,33]	
**60**	phillyrin (forsythin)	UFF, RFF	[6,17,33]	
**61**	caffeoyl phillygenin	N.M.	[22]	
**62**	(+) epipinoresinol	RFF	[33]	
**63**	7′-epi-8-hydroxypinoresinol	N.M.	[32]	
**64**	(+) epipinoresinol-4-*O*-β-d-glucoside	N.M.	[34]	
**65**	(+)-8-hydroxyepipinoresinol-4-*O*-β-d-glucopyranoside	N.M.	[34]	
**66**	(+) epipinoresinol-4′-*O*-β-d-glucoside	N.M.	[34]	
**67**	forsythialanside E	N.M.	[24]	
**68**	pinoresinol	N.M.	[32]	
**69**	(+) pinoresinol-β-d-glucoside	N.M.	[35]	
**70**	(+) pinoresinol monomethyl ether-β-d-glucoside	N.M.	[35]	
**71**	pinoresinol diglucoside	N.M.	[22]	
**72**	caffeoyl pinoresinol	N.M.	[22]	
**73**	(+)-1-hydroxypinordsinol/8-hydroxypinoresinol	N.M.	[19,32]	
**74**	(+)-1-hydroxypinordsinol-4′-*O*-β-d-glucoside	N.M.	[19]	
**75**	(+)-1-hydroxypinordsinol-4′-*O*-β-d-glucoside	N.M.	[19]	
**76**	3′,4′,5′-trimethoxy-4′′-hydroxyllignan *O*-glucoside	N.M.	[22]	
**77**	syringaresinol-4-*O*-β-d-glucoside	N.M.	[23]	
**78**	isolariciresinol	UFF, RFF	[15,33,36]	
**79**	isolariciresinol-4-*O*-β-d-glucopyranoside	RFF	[36]	
**80**	isolariciresinol-9′-*O*-β-d-glucopyranoside	RFF	[36]	
**81**	isoolivil	RFF	[36]	
**82**	cedrusin	N.M.	[32]	
**83**	glochidioboside	N.M.	[34]	
**84**	forsythialanside C	N.M.	[23]	
**85**	forsythialanside D	N.M.	[23]	
**86**	dihydrodehydrodiconiferyl alcohol-4-*O*-β-d-glucoside	N.M.	[23]	
**87**	icariside E4	N.M.	[23]	
**88**	forsythialan A	N.M.	[37]	
**89**	forsythialan B	N.M.	[37]	
**90**	*rel*-(7*R*,8′*R*,8*S*)-forsythialan C	N.M.	[38]	
**91**	*rel*-(7*R*,8′*R*,8*R*)-forsythialan C	N.M.	[38]	
**92**	forsythialanside A	N.M.	[23]	
**93**	forsythialanside B	N.M.	[23]	
**94**	olivil	UFF	[17,32]	
**95**	forsythiayanoside B	N.M.	[34]	
**96**	forsythiayanoside A	N.M.	[34]	
**97**	3-furanone-2-(3-methoxy-4-hydroxyphenyl)-4-veratryl	N.M.	[22]	
**98**	lariciresinol	N.M.	[32]	
**99**	lariciresinol-4-*O*-β-d-glucoside	N.M.	[24]	
**100**	lariciresinol-4′-*O*-β-d-glucoside	N.M.	[24]	
**101**	benzenebutanoic acid	N.M	[39]	
**Aliphatic C6-C2 alcohols**				
**102**	isorengyol	N.M.	[40]	
**103**	rengyol	UFF	[6,20,40]	
**104**	suspenol	N.M.	[41]	
**105**	rengyolester	N.M.	[42]	
**106**	rengyoside A	N.M.	[29]	
**107**	rengyoside C	N.M.	[29]	
**108**	rengynic acid	N.M.	[14,43]	
**109**	rengynic acid-1′-*O*-β-d-glucopyranoside	N.M.	[44]	
**110**	rengyolone (halleridone)	N.M.	[20,29]	
**111**	rengyoxide	N.M.	[20]	
**112**	rengyoside B	N.M.	[29]	
**113**	cornoside	RFF	[6,23]	
**114**	forsythenside A	N.M.	[23,45]	
**115**	forsythenside B	N.M.	[45]	
**116**	forsythenside F	N.M.	[46]	
**117**	forsythenside H	N.M.	[23]	
**118**	forsythenside G	N.M.	[23]	
**119**	forsythenside I	N.M.	[23]	
**120**	forsythenside J	N.M.	[23]	
**Iridoids**				
**121**	adoxosidic acid	UFF, RFF	[6]	
**122**	adoxosidic acid 10-*p*-hydroxyphenylacetate	N.M.	[38]	
**Diterpenoids**				
**123**	3β-hydroxylabda-8(17), 13(*E*)-dien-15-oic acid	N.M.	[47]	
**124**	3β-hydroxyanticopalic acid	N.M.	[48]	
**125**	agatholic acid	N.M.	[48]	
**126**	3-oxoanticopalic acid	N.M.	[38]	
**127**	19-hydroxylabda-8(17),13(*Z*)-dien-15-oic acid	N.M.	[38]	
**128**	19-hydroxylabda-8(17),13(*E*)dien-15-oic acid	N.M.	[38]	
**129**	19-formyllabda-8(17),13(*E*)-dien-15-oic acid	N.M.	[38]	
**130**	19-formyllabda-8(17),13(*Z*)-dien-15-oic acid	N.M.	[38]	
**131**	labda-8(17),13(*Z*)-dien-15,18-dioic acid	N.M.	[38]	
**132**	labda-8(17),13(*Z*)-diene-15,19dioic acid	N.M.	[38]	
**133**	labda-8(17),13(*E*)-diene-15,19-dioic acid	N.M.	[38]	
**134**	dehydropinifolic acid	N.M.	[38]	
**135**	haplopappic acid	N.M.	[38]	
**136**	18-hydroxy-7-oxolabda-8(9),13(*E*)-dien-15-oic acid	N.M.	[38]	
**137**	17,19-dihydroxylabda-7(8),13(*E*)-dien-15-oic acid	N.M.	[38]	
**138**	forsythidin A	N.M.	[38]	
**139**	3β-hydroxy-12,13(*E*)-biformene	N.M.	[38]	
**140**	3β-hydroxy-12,13(*Z*)-biformene	N.M.	[38]	
**141**	19-hydroxy-8(17)(*E*)-13-labdadien-15-oate	N.M.	[38]	
**Triterpenoids**				
**142**	ocotillone	N.M.	[49]	
**143**	ocotillol monoacetate	N.M.	[49]	
**144**	garcinielliptone Q	N.M.	[38]	
**145**	3β-acetyl-20,25-epoxydammarane-24α-ol	N.M.	[50]	
**146**	3β-acetyl-20,25-epoxydammarane-24β-ol	N.M.	[50]	
**147**	dammar-24-en-3β-acetoxy-20-ol	N.M.	[38,47,51]	
**148**	3β-acetoxy-25methoxydammar-23-en-20β-ol	N.M.	[38]	
**149**	3β-acetoxyl-20S,24R-dammarane-25-ene-24-hydroperoxy-20-ol	N.M.	[47]	
**150**	cabralea lactone 3-acetate	N.M.	[47]	
**151**	cabralea lactone 3-acetate 24-methyl ether	N.M.	[38]	
**152**	3-acetylisofouquierol	N.M.	[47]	
**153**	oleanolic acid	RFF	[33,52]	
**154**	3β-acetyloleanolic acid	N.M.	[48]	
**155**	β-amyrin acetate	N.M.	[47]	
**156**	ursolic acid	RFF	[33]	
**157**	2α,3α-hydroxyursolic acid	N.M.	[53]	
**158**	2α,23-hydroxyursolic acid	RFF	[33]	
**159**	3β-acetoxy-20α-hydroxyursan-28-oic acid	N.M.	[48]	
**160**	betulinic acid	RFF	[33,52]	
**161**	3β-acetylbetulinic acid	N.M.	[54]	
**162**	2α-hydroxybetulinic acid	RFF	[33]	
**163**	ambrolic acid	N.M.	[51,55]	
**164**	morolic acid	N.M.	[47]	
**165**	3β-acetoxyolean-12-en-28-oic acid	N.M.	[38]	
**166**	alphitolic acid	N.M.	[38]	
**167**	onjisaponin F	N.M.	[53]	
**168**	onjisaponin G	N.M.	[53]	
**Sterols**				
**169**	β-sitosterol	N.M.	[56]	
**170**	daucosterol	N.M.	[57]	
**171**	taraxasterol acetate	N.M.	[48]	
**172**	stigmasterol	N.M.	[48]	
**173**	*ψ*-taraxasterol	N.M.	[48]	
**174**	(6′-*O*-palmitoyl)-sitosterol-3-*O*-β-d-glucoside	N.M.	[49]	
**Flavonoids**				
**175**	rutin	UFF, RFF	[6,22,58]	
**176**	rutin-*O*-hexoside	N.M.	[22]	
**177**	quercetin	UFF, RFF	[58]	
**178**	quercetin-*O*-rhamnosyl hexoside	N.M.	[22]	
**179**	trimethoxyquercetin-*O*-feruloyl rhamnoside	N.M.	[22]	
**180**	isorhamnetin	N.M	[59]	
**181**	kaempferfol	N.M.	[22]	
**182**	hyperin	N.M.	[18]	
**183**	kaempferol dirhamnoside	N.M.	[22]	
**184**	kaempferol-*O*-rhamnosylhexoside	N.M.	[22]	
**185**	kaempferol-3-*O*-β-d-glucopyranoside-7-*O*-α-l-rhamnopyranoside	N.M.	[11]	
**186**	kaempferol-3-*O*-β-d-(2″-*O*-β-d-glucopyranosyl-6″*O*-α-l-rhamno-pyranosyl)glucopyranoside	N.M.	[11]	
**187**	wogonin-7-*O*-glcoside	N.M.	[60]	
**188**	baicalin	UFF, RFF	[58]	
**189**	hesperidin	N.M.	[18]	
**190**	forsythoneoside A	N.M.	[11]	
**191**	forsythoneoside B	N.M.	[11]	
**192**	forsythoneoside C	N.M.	[11]	
**193**	forsythoneoside D	N.M.	[11]	
**Volatiles**				
**194**	β-pinene	N.M.	[61]	
**195**	myrtenol	N.M.	[61]	
**196**	(+)-α-pinene	N.M.	[61]	
**197**	(−)-trans-pinocarveol	N.M.	[61]	
**198**	sabinene	N.M.	[61]	
**199**	pinocarvone	N.M.	[61]	
**200**	(−)-terpinen-4-ol	N.M.	[61]	
**201**	dipentene	N.M.	[61]	
**202**	camphene	N.M.	[61]	
**203**	myrcene	N.M.	[61]	
**204**	α-terpinene	N.M.	[61]	
**205**	*O*-cymene	N.M.	[61]	
**206**	eucalyptol (1,8-cineole)	N.M.	[61]	
**207**	γ-terpinene	N.M.	[61]	
**208**	campholenic aldehyde	N.M.	[61]	
**209**	(*S*)-*cis*-verbenol	N.M.	[61]	
**210**	2,5-cyclooctadien-1-ol	N.M.	[61]	
**211**	(1*S*)-(−)-verbenone	N.M.	[61]	
**212**	α-pinene	N.M.	[61]	
**213**	β-phellandrene	N.M.	[62]	
**214**	(+)-carene	N.M.	[62]	
**215**	α-terpinolene	N.M.	[62]	
**216**	1,4-cyclohexadiene	N.M.	[62]	
**217**	4-carvomenthenol	N.M.	[62]	
**218**	(±)-α-terpinel	N.M.	[62]	
**219**	(−)-myrtenal	N.M.	[62]	
**220**	2-methyl-5-(1-methylethenyl)cyclohexanol	N.M.	[62]	
**221**	estragole	N.M.	[62]	
**222**	1-hexanol	N.M.	[63]	
**223**	(−)-β-pinene	N.M.	[63]	
**224**	(+)-4-carene	N.M.	[63]	
**225**	linalool	N.M.	[64]	
**226**	*trans*-carveol	N.M.	[64]	
**227**	*p*-cymen-8-ol	N.M.	[64]	
**228**	trans-nerolidol	N.M.	[64]	
**229**	camphor	N.M.	[64]	
**230**	β-ocimene	N.M.	[64]	
**231**	germacrene D	UFF	[65]	
**232**	α-cubebene	UFF	[65]	
**233**	bornyl acetate	UFF	[65]	
**234**	*cis*-piperitol	UFF	[65]	
**235**	α-pinocarvone	UFF	[65]	
**236**	α-terpineol	UFF	[65]	
**237**	ocimene	UFF	[62,65]	
**238**	α-phellandrene	UFF	[65]	
**239**	nutmeg aldehyde	RFF	[65]	
**240**	(-)-alloaromadendren	RFF	[65]	
**241**	cumene formaldehyde	RFF	[65]	
**242**	3-cyclohexene-1-methanol	RFF	[65]	
**243**	4-methylene-1-cyclohexanone	RFF	[65]	
**244**	*p*-cymene	UFF	[66]	
**245**	limonene	UFF	[66]	
**Alkaloids**				
**246**	rutaecarpine	N.M.	[57]	
**247**	suspensine A	UFF	[67]	
**248**	(−)-egenine	UFF	[67]	
**249**	(−)-7′-*O*-methylegenine	UFF	[67]	
**250**	(−)-bicuculline	UFF	[67]	
**251**	bis-2-(4-aminophenyl)ethyl-β-d-glucopyranoside	N.M.	[68]	
**252**	choline	UFF, RFF	[6]	
**Organic acids**				
**253**	palmitic acid	N.M.	[56]	
**254**	stearic acid	N.M.	[56]	
**255**	succinic acid	UFF, RFF	[6]	
**256**	suspenolic acid	N.M.	[45]	
**257**	2-furancarboxylic acid	N.M.	[48]	
**258**	chlorogenic acid	N.M.	[18]	
**259**	anchoic acid	UFF, RFF	[58]	
**260**	4-hydroxy-4-isopropylcyclohex-1-enecarboxylic acid	UFF, RFF	[58]	
**261**	*p*-coumaric acid	UFF, RFF	[58]	
**262**	protocatechuic acid	seeds	[69]	
**263**	vanillic acid	N.M.	[70]	
**264**	*p*-hydroxybenzoic acid	N.M.	[48]	
**265**	benzoic acid	N.M.	[48]	
**266**	3,4-dimethoxybenzoic acid	N.M.	[48]	
**267**	syringic acid	N.M.	[48]	
**268**	caffeic acid	N.M.	[70]	
**269**	*trans*-coumaric acid	N.M.	[48]	
**270**	*trans*-ferulic acid	N.M.	[48]	
**271**	caffeic acid methyl ester	RFF	[36]	
**272**	*p*-hydroxybenylacetic acid	N.M.	[70]	
**273**	tannic acid	N.M.	[71]	
**274**	gallic acid	RFF	[6]	
**275**	3-hydroxybutyric acid	UFF	[6]	
**276**	acetic acid	UFF, RFF	[6]	
**277**	pyruvic acid	UFF, RFF	[6]	
**278**	malic acid	UFF, RFF	[6]	
**279**	fumaric acid	UFF	[6]	
**280**	formic acid	UFF	[6]	
**Amino acids**				
**281**	isoleucine	UFF	[6]	
**282**	leucine	UFF	[6]	
**283**	valine	UFF, RFF	[6]	
**284**	threonine	UFF	[6]	
**285**	alanine	UFF	[6]	
**286**	phenylalanine	RFF	[6]	
**Sugar derivatives**				
**287**	β-xylose	UFF, RFF	[6]	
**288**	β-glucose	UFF	[6]	
**289**	α-glucose	UFF, RFF	[6]	
**290**	raffinose	UFF	[6]	
**291**	sucrose	RFF	[6]	
**292**	l-rhamnose	RFF	[36]	
**293**	lactose	N.M.	[72]	
**294**	erythritol	N.M.	[60]	
**295**	[4]-α-d-GalpA-(1→2]_7_-[4]-α-d-GalpA-(1→2)-α-l-Rhap-(1→2]_2_	N.M.	[73]	
**Allylbenzene glycosides**				
**296**	forsythenside L	N.M.	[23]	
**297**	sasanquin	N.M.	[23]	
**Other compounds**				
**298**	forsythiayanoside D	UFF	[30]	
**299**	(6*S*,9*R*)-roseoside	N.M.	[48]	
**300**	swertiamacroside	N.M.	[74]	
**301**	2,3,5,6-tetrahydrojacaranone-4-*O*-β-d-glucopyranoside	N.M.	[14]	
**302**	labda-8(17),13(*E*)-dien-15,18-dioic acid 15-methyl ester	N.M.	[48]	
**303**	β-carotene-5,6-epoxide	N.M.	[72]	
**304**	mutatochrome	N.M.	[72]	
**305**	neoxanthin	N.M.	[72]	
**306**	1-oxo-4-hydroxy-2(3)-en-4-ethylcyclohexa-5,8-olide	N.M.	[38]	
**307**	esculetin	N.M.	[48]	
**308**	6,7-dimethoxycouma	N.M.	[53]	
**309**	hydroxytyrosol	N.M.	[48]	
**310**	*p*-tyrosol	N.M.	[48]	
**311**	4-hydroxybenylacetic acid methyl ester	RFF	[36]	
**312**	4-caffeoylrutinose	N.M.	[20]	
**313**	protocatechualdehyde	N.M.	[48]	
**314**	*p*-hydroxyphenylethanol	UFF, RFF	[58]	
**315**	*p*-hydroxybenzylalcohol	UFF, RFF	[58]	
**316**	*n*-hentriacontane	UFF	[75]	
**317**	2,3-dihydroxymethyl-4-(3′,4′-dimethoxyphenyl)-*γ*-butyrolactone	N.M.	[57]	
**318**	methyl-α-d-glucopyranoside	N.M.	[48]	
**319**	forsythenin	N.M.	[49]	
**320**	4-*O*-demethylforsythenin	N.M.	[38]	
**321**	salicifoliol	N.M.	[38]	

N.M.: Compounds that have not been specifically mentioned from UFF or RFF.

**Table 2:** Quantitative analysis for the quality control of *Forsythiae Fructus.*

Analytes	Method	Results	Reference
Phillyrin	LC-MS	The contents of phillyrin in *Forsythiae Fructus* and three medicinal preparations (Xiao′erqingyan granules, Niuhuangshangqing pills, Yinqiao tablets) were 1.30, 0.48, 3.36, 0.35 mg/g, respectively	[78]
Phillyrin	HPLC	The contents of phillyrin in *Forsythiae Fructus* from ten habitats were from 0.72 to 3.54 mg/g, indicating the influence of habitat on the quality of *Forsythiae Fructus*.	[79]
Phillyrin Forsythoside A	HPLC	In four batches of UFF, the contents of phillyrin and forsythoside A were 0.73–2.16% and 0.85–1.56%, respectively. In eleven batches of RFF, the contents of phillyrin and forsythoside A were 0.57–2.50% and 0.33–0.76%, respectively.	[80]
Phillyrin, Forsythoside A	HPLC	The contents of phillyrin and forsythoside A from three batches were 3.08–4.35 mg/g and 15.89–20.76 mg/g, respectively.	[81]
Rutin Forsythin	CE-ED	The contents of rutin and forsythin in *Forsythiae Fructus* were 2.03 mg/g and 2.95 mg/g, respectively.	[82]
Forsythoside A Rutin Phillyrin	HPLC	In UFF from different harvesting times, the contents of forsythoside A, rutin and phillyrin were 3.87–8.72%, 0.05–0.36% and 0.10–0.63%, respectively, which reached a peak in early July.	[83]
Forsythoside A, Phillyrin, Phillygenin	HPLC	In three batches of UFF, the average contents of forsythoside A, phillyrin and phillygenin were 3.3385, 0.2934 and 0.4873 mg/g, respectively. In the RFF, the average contents were 0.3129, 0.2228 and 0.9258 mg/g, respectively.	[84]
Rutin Forsythoside A Phillyrin	HPLC-PDA	The contents of rutin, forsythoside A and phillyrin in three batches of RFF were linear in the range of 0.1–2.0, 0.12–2.4 and 0.05–1.0 μg/g, respectively.	[85]
Forsythoside A Rutin Forsythin	HPLC-ESI-MS	In UFF, the contents of forsythoside A, rutin and forsythin were 3.783%, 0.105% and 0.365%, respectively. In RFF, the contents were 0.257%, 0.167% and 0.043%, respectively.	[86]
(+)-Pinoresinol-β-d-glucoside, Forsythoside A, Phillyrin Phillygenin	HPLC-PDA	In nineteen batches of UFF, the contents of (+)-pinoresinol-β-d-glucoside, forsythoside A, phillyrin and phillygenin were 3.95–6.14%, 9.15–15.71%, 0.80–1.64% and 0.70–2.10%, respectively. In nineteen batches of RFF, the contents were 3.76–5.55%, 5.91–10.59%, 0.45–1.27% and 1.40–2.00%, respectively. Apart from the harvest times, the plant origins, manufacturing methods and storage conditions also played a role in the variation of the contents of the active components.	[87]
Total flavonoids, Forsythoside A , Rutin, Quercetin	HPLC	In UFF, the contents of total flavonoids, forsythin, forsythoside A, rutin and quercetin were 1.362%, 29.95 ± 0.06 mg/g, 64.0325 ± 0.03 mg/g, 2.6075 ± 0.02 mg/g and almost 0 mg/g, respectively. In RFF, the contents of them were 1.099%, 22.975 ± 0.04 mg/g, 58.3325 ± 0.03 mg/g, 0.57075 ± 0.01 mg/g and 0.0209 ± 0.07 mg/g, respectively.	[88]
Cafferic acid, Forsythoside A, Forsythoside B, Rutin, Hyperin, Forsythin Arctigenin	RP-HPLC	The contents of cafferic acid, forsythoside A, forsythoside B, rutin, hyperin, forsythin and arctigenin in *Forsythiae Fructus* from six origins were 3.377–7.457 mg/g, 14.06–88.00 mg/g, 1.325–3.196 mg/g, 0.2682–3.1470 mg/g, 0.4109–0.7008 mg/g, 2.128–5.226 mg/g and 0.7437–3.6720 mg/g, respectively.	[89]
Chlorogenic acid, R-suspensaside, S-suspensaside, S-suspensaside methyl ether, Forsythoside, (+)-Pinoresinol-β-d-glucoside, (+)-Epipinoresinol-4′-*O*-glucoside, Matairesinol-4′-*O*-glucoside, rutin, Hesperidin, Hyperin, Phillyrin, Phillygenin, (+)-Epipinoresinol	LC-ESI-MS	The fourteen compounds from twelve batches of *Forsythiae Fructus* from nine regions were quantified and were present at 0.0004–0.0068%, 0.0098–0.0795%, 0.0167–0.1482%, 0.0100–0.4904%, 0.2076–0.8693%, 0.0086–0.2044%, 0.0073–0.1720%, 0.0070–0.0724%, 0.0742–0.2226%, 0.0041–0.0257%, 0.0010–0.0059%, 0.0200–0.4236%, 0.0448–0.1020% and 0.0024–0.1231%, respectively.	[18]
R-suspensaside, S-suspensaside methyl ether, (+)-Pinoresinol-β-d-glucoside, Forsythoside A, (+)-Epipinoresinol-4′-*O*-glucoside, Suspensaside A, Rutin, Phillyrin, Pinoresinol, (+)-Epipinoresinol and Phillygenin	HPLC-DAD	The levels of twelve constituents varied from 16.86 to 74.55 mg/g; rutin is the most stable, with only three-fold variation in the detected thirty-three samples. As the main compound, the contents of forsythoside A ranged from 5.15 to 55.78 mg/g.	[90]
Forsythoside E, Forsythoside A , Suspensaside A, Rutin, Baicalin, Quercetin, Phillyrin, (+)-Epipinoresinol, (+)-Pinoresinol-4-*O*-β-d-glucoside (+)-Epipinoresinol-4-O-β-d-glucoside, Chlorogenic acid, *p*-Hydroxybenzoic acid, *p*-Coumaric acid, Anchoic acid 4-Hydroxy-4-isopropylcyclohex-1-enecarboxylic acid, *p*-Hydroxyphenyl-ethanol, *p*-Hydroxybenzylalcohol	HPLC–ESI-MS/MS	In the UFF, the contents of forsythoside A, phillyrin, (+)-epipinoresinol, (+)-epipinoresinol-4-*O*-β-d-glucoside, (+)-pinoresinol-4-*O*-β-d-glucoside were 31.1–41.7, 10.8–12.7, 11.1–21.0, 9.1–16.4, 5.2–14.4 mg/g, respectively. In the RFF, the contents of them were 6.7–8.5, 0.8–5.4, 1.6–6.4, 2.2–5.8, 1.2–4.8 mg/g, respectively. Moreover, total contents of flavonoids in the UFF were higher than in the RFF, while those of phenolic acids were on the contrary. Contents of the aliphatic acids and terpenoids were not significantly different between the UFF and the RFF.	[58]
α-pinene, Camphene, β-Pinene, Myrcene, *p*-Cymene, Limonene α-Terpineol	GC	In the UFF from sixteen batches, the contents of α-pinene, camphene, β-pinene, myrcene, *p*-cymene, limonene and α-terpineol were 0.102–0.337%, 0.004–0.018%, 0.342–1.024%, 0.008–0.024%, 0.006–0.032%, 0.003–0.029% and 0.003–0.017%, respectively.	[66]
α-Pinene β-Pinene	GC	In the UFF, the contents of α-pinene and β-pinene were 0.192–0.300% and 0.556–0.934%, while the contents of them were 0.075% and 0.240% in the RFF.	[91]
(+)-Pinoresinol-β-d-glucoside, Matairesinol-4′-*O*-glucoside, Hyperin, Phillyrin, Phillygenin	HPLC-ESI-MS/MS	The contents of (+)-pinoresinol-β-d-glucoside, matairesinol-4′-*O*-glucoside, hyperin, phillyrin and phillygenin in the 75% methanol extract of *Forsythiae Fructus* were 227.00, 70.80, 2.67, 225.20 and 106.10 mg/mL, respectively.	[31]

**Table 3:** Pharmacological effects of *Forsythiae Fructus.*

Models	Constituent/Extract	Mechanism	Reference
**Anti-inflammatory Activity**			
LPS-induced liver injury in rats	Ethanol extract	The extract inhibited generation of ROS, MDA, TNF-α, IL-1β and IL-6 in serum and liver via activation of Nrf2-mediated antioxidation and inhibition of NF-κB-mediated inflammatory response.	[92]
LPS-stimulated RAW 264.7 cells	Ethyl acetate fraction of the ethanol extract	The extract at 12.5–200 μg/mL inhibited expression of COX-2, thus decreasing the levels of ROS, NO and PGE_2_ does-dependently.	[93]
LPS-stimulated BV-2 microglial cells	Aqueous extract Forsythin	The extract at 1 μg/mL inhibited the MAPK pathway and down-regulated NO biosynthesis-related genes. Forsythin at 50–200 μg/mL significantly suppressed the production of NO and decreased iNOS and TRL4 protein expression in a dose dependent manner.	[94,95]
Soybean β-conglycinin-stimulated weaned piglets	Methanol extract	The methanol extract (100 mg/kg) reduced the levels of anaphylactic antibodies, mast cell degranulation, histamine release, T lymphocyte proliferation and IL-4 synthesis and improved intestinal microbial flora.	[96]
*Dermatophagoides farinae*-induced atopic dermatitis in NC/Nga mice	Ethanol extract Forsythoside A, Phillyrin, Pinoresinol, Phylligenin	The extract (25, 50, 100, 200 and 400 μg/mL) suppressed expression of chemokines (TARC, MDC and RANTES), adhension molecules (ICAM-1 and VCAM-1) and inflammatory factors (TNF-α and IL-4) in ear tissues. It could also inhibit the production of chemokines in keratinocytes. Further study revealed that forsythoside A, phillyrin, pinoresinol and phylligenin may be the active constituents for the therapy of atopic dermatitis.	[97]
Carrageenan-induced rats	Ethanol extract	The extract (5 g/kg) alleviated carrageenan-induced paw edema in rats, probably by increasing the production of COX-2 and decreasing the expression of PGE_2_, PGD_2_, 6-keto-PGF1α and TXB_2_.	[98]
Xylene-stimulated mice Acetic acid-stimulated mice Carrageenan-induced rats Oleic acid-stimulated rats	Volatiles	Volatiles inhibited the ear-swelling induced by xylene at 0.12 and 0.24 mL/kg, withstood the hyperfunction of celiac capillary permeability induced by acetic acid at 0.24 mL/kg, alleviated rats paw edema induced by carrageenan at 0.12 and 0.24 mL/kg, inhibited pleuritis induced by carrageenan at 0.24 mL/kg and decreased acute lung injury induced by oleic acid at 0.12 and 0.24 mL/kg.	[99]
**Anti-inflammatory Activity**			
LPS/D-galactosamine-induced acute liver injury mice	Forsythoside A	Forsythoside A (15, 30 and 60 mg/kg) decreased the serum levels of ALT, AST and TNF-α, increased expression of Nrf2 and heme oxygenase-1 and inhibited NF-κB activation, thus protecting against LPS/D-galactosamine-induced acute liver injury.	[100]
LPS-stimulated RAW264.7 cells	Forsythin	Forsythin (25, 50, 100, 150 and 200 μg/mL) inhibited the production of ROS, IL-6, IL-1β, TNF-α, NO, PGE_2_, iNOS and COX-2 in a dose dependent manner by suppressing JAK-STAT and p38 MAPK signaling pathway.	[101]
LPS-stimulated RAW264.7 cells	Forsythoside A	Treatment with forsythoside A in LPS-stimulated RAW264.7 cells reduced the secretion of TNF-α, IL-6 and NO via inhibition of HMGB1/TLR4/NF-κB pathaway.	[102]
LPS-induced acute lung injury male BALB/c mice	Phillyrin	Phillyrin (20 mg/kg) pretreatment significantly decreased the production of IL-1β, IL-6, TNF-α and the concentration of myeloperoxidase in lung tissues via inhibition of MAPK and NF-κB pathways.	[103]
LPS-stimulated RAW264.7 cells	Arctiin	Arctiin (12.5, 25, 50 and 100 μg/mL) inhibited NF-κB pathway, thus reducing the production of IL-1β, IL-6, TNF-α and PGE_2_ in a dose dependent manner, as well as expression of co-stimulatory molecules (B7-1 and B7-2).	[104]
LPS-stimulated BEAS-2B cells	90% Forsythoside A extracts	Forsythoside A extracts (25, 50 and 100 μg/mL) significantly reduced the production of NO in a dose-dependent manner and the level of intracellular ROS in a dose-effect manner.	[105]
Bursa of Fabricius of chickens	Forsythoside A	Forsythoside A (30 and 60 mg/kg) suppressed the NF-κB-iNOS-NO signaling pathway to reduce the production of IL-6, IL-1β, TNF-α and COX-2.	[76]
Allergic dermatitis in NC/Nga mice	Ethanol extract Matairesinol	In vitro, the *Forsythiae Fructus* ethanol extracts at 200 μg/mL inhibited histamine to release from mast cells. Further study revealed that matairesinol suppressed inflammatory cell infiltration, IL-4 and IFN-γ mRNA expression and lowered IgE levels in vivo.	[106]
**Anti-inflammatory Activity**			
COPD mice	Forsythoside A	Forsythoside A (15, 30 and 60 mg/kg) suppressed the production of IL-1β, IL-6, TNF-α and NO and reversed cigarette smoke induced GSH/GSSG ratio, which were related to activation of Nrf2 dose-dependently and inhibition of NF-κB.	[107]
Male C57LB/6 mice	Forsythin	As a selective inhibitor of PDE4, forsythin significantly decreased the levels of IL-1β, IL-6 and TNF in LPS/H1N1 influenza-induced lung injury and sepsis in vivo. Moreover, authors took it as a lead compound and developed three other PDE4 inhibitors with higher activities.	[108]
Male Sprague-Dawley rats RAW 264.7 cells	Arctigenin	Arctigenin (0.1–1.0 mg/ear) significantly decreased myeloperoxidase and eosinophil peroxidase activities in the arachidonic acid (AA) induced edematous tissues homogenate and silica-induced ROS production in the RAW 264.7 cell line at 0.1–10 μM, probably by inhibiting the release or production of AA metabolites and free radicals.	[109]
LPS-stimulated BV2 microglia cells nd primary microglia cells	Forsythoside A	Forsythoside A at 2.5, 5 and 10 μg/mL inhibited the production of TNF-α, IL-1β, NO and PGE_2_ via inhibiting NF-κB and activating Nrf2/HO-1 signaling pathway.	[110]
PAF-stimulated rat polymorphonuclear leukocytes	Suspensine A, 7′-*O*-methylegenine, (−)-Egenine, (−)-Bicuculline	The four alkaloids at 10 μM inhibited the release of β-glucuronidase from polymorphonuclear leukocytes of rats with the rates of 39.6%, 37.7%, 36.5% and 34.8%, respectively.	[67]
*Staphylococcus aureus (S. aureus)*-stimulated monocyte-macrophage	Forsythin	Forsythin at 50 mg/L significantly decreased expression of IL-8, TNF-α, IL-6 and at 100 mg/L also decreased expression of macrophage colony stimulating factor-1 (MCSF-1) dose-dependently.	[111]
**Antibacterial Activity**			
*Escherichia coli (E. coli)* *Staphylococcus aureus (S. aureus)*	Essential oil	The essential oil changed the permeability and integrity of the cell membrane, leading to leakage of nucleic acids and proteins with MIC values of 3.13 and 1.56 mg/mL for *E. coli* and *S. aureus*, respectively.	[112]
*Pneumococcus, Escherichia coli (E. coli), S. aureus*, *Haemophilus influenza, a beta-group Streptococcus, Yersinia enterocxolitica, Klebsiella pneumonia, F‘s dysentery bacillus, Salmonella typhi, Pseudomonas aeruginosa*	Essential oil	The essential oil showed antibacterial activity against these ten bacteria. Particularly, β-pinene and the oil after chromatography showed a better inhibitory effect on the other bacteria, except *Yersinia enterocolitica* and *Klebsiella pneumonia.*	[113]
*Escherichia coli (E. coli)* (BCRC-11634)	3β-Acetoxyl-20α-hydroxyursan-28-oic acid β-Amyrin acetate, Betulinic acid ψ-Taraxasterol, 3β-Hydroxyanticopalic acid Agatholic acid, Phillyrin	The seven compounds showed antibacterial effect with MIC values of 4.55, 5.00, 1.20, 1.20, 3.42, 2.62 and 3.94 mg/mL, respectively.	[48]
*Staphylococcus aureus* (*S. aureus*)	Ethanol extract	The extract inhibited secretion of α-hemolysin in the range of 16–128 mg/L dose-dependently.	[114]
*Escherichia coli (E. coli)*, *Pseudomonas aeruginosa*, *Staphylococcus aureus* (*S. aureus*)	Isoforsythoside A Forsythoside A	The MIC of isoforsythoside A for *E. coli, Pseudomonas aeruginosa* and *S. aureus* were 40.83, 40.83 and 81.66 μg/mL, respectively, and those of forsythoside A were 38.33, 38.33 and 76.67 μg/mL, respectively.	[27]
*Esherichia coli (E. coli)* K88, *Staphylococcus aureus* (*S. aureus*) *Salmonella enteric* 34R99	Methanol extract	The *Forsythiae Fructus* methanol extracts protected against *E. coli* K88, *S. aureus* and *Salmonella enteric* 34R99 with minimum concentrations of 25.00, 12.50 and 1.56 mg/mL, respectively.	[115]
*Helicobacter pylori*	Betulinic acid Oleanolic acid	The *Forsythiae Fructus* ethanol extracts strongly (82%) inhibited urease activity of *Helicobacter pylori.* Further study revealed that the active compounds were betulinic acid and oleanolic acid.	[52]
*Acinetobacter baumannii*	Aqueous extract	The aqueous decoction of *Forsythiae Fructus* inhibited the active efflux pump and induced mutations in the nucleotide sequence of the adeb gene at 2.5 and 5 mg/mL.	[116]
**Antiviral Activity**			
H1N1-infected MDCK cells	80% Ethanol extract	The 80% ethanol extract of *Forsythiae Fructus* exhibited an inhibitory effect on H1N1 in a dose-dependent manner at the concentration of 1:512 to 1:8192 mg/mL.	[8]
H1N1-infected human bronchial epithelial cell line A549	95% Ethanol extract 50% Ethanol extract Aqueous extract	95% Ethanol extract, 50% ethanol extract and aqueous extract exhibited inhibitory effect on RANTES secretion with IC_50_ values of 42 ± 6, 117 ± 15 and 232 ± 28 μg/mL, respectively. Moreover, 95% ethanol extract displayed dual regulatory effects on MCP-1 production, while 50% ethanol extract and aqueous extract increased MCP-1 production by 1.4–3.3 and 2.6–3.7 times, respectively.	[117]
C57BL/6j mice	Forsythoside A	Forsythoside A (0.4 μg/mL) inhibited influenza A virus replication by suppressing the expression of TLR7, MyD88, TRAF6, IRAK4 and NF-κB p65 mRNA in vivo.	[77]
male BALB/C mice	Phillyrin	Phillyrin at a dose of 20 mg/kg/day protected against influenza A shown by the reduction of lung index, viral titers, IL-6 levels, expression of hemagglutinin protein and the alleviated lung tissue damage.	[118]
Influenza A transfected-HeLa cells	Phillyrin	Phillyrin significantly decreased the gene expression of IAV nucleoprotein.	[119]
PRRSV-infected Marc-145 cells	Forsythoside A	Forsythoside A inhibited porcine reproductive and respiratory syndrome virus (PRRSV) RNA synthesis and promoted secretion of IFN-α. The sterilization rate reached 80% at a concentration of 60 μg/mL.	[120]
RSV-infected MDCK cells and Hep-2 cells	Calceolarioside B Forsythoside A	Calceolarioside B and forsythoside A exhibited EC_50_ values of 3.43 and 6.72 μM for RSV, respectively.	[23]
RSV	Rengynic acid	Rengynic acid exhibited an anti-RSV effect with EC_50_ and MIC values of 9.9 and 41.66 μg/mL, respectively.	[43]
IBV-infected primary chicken embryo kidney cells	Forsythoside A	Forsythoside A pretreatment at a dose of 0.64 mM had a direct virucidal effect on IBV, but it had no effect on IBV-infected cells.	[121]
IBV-infected HD11 cells	Forsythoside A	Forsythoside A (10 and 20 μM) exhibited an antiviral effect by significantly increasing expression of intracellular receptors (MDA5, LGP2 and NLRC5) and antiviral gene (IRF7, IFN-α, IFN-β) mRNA.	[122]
Antioxidant Activity			
DPPH	Isoforsythoside A	Isoforsythoside A exhibited antioxidant activity with an EC_50_ value of 2.74 μg/mL and Vc exhibited an IC_50_ of 4.38 μg/mL in the DPPH assay.	[27]
DPPH and superoxide anion	Polysaccharides	*Forsythiae Fructus* polysaccharides showed significant scavenging capacity on the DPPH and superoxide anion with IC_50_ values of 0.08 and 2.0 mg/mL, respectively.	[123]
DPPH in vitro and diquat-stimulated male Sprague Dawley rats in vivo	CH_2_Cl_2_ fraction of ethanol extract Forsythoside A Forsythialan A Phillygenin Phillyrin	The CH_2_Cl_2_ fraction of ethanol extract (25, 50 and 100 mg/kg) reduced expression of TNF-α, IL-1β, IL-6, MDA and increased the activities of SOD, GSH-Px, GSH. Forsythoside A, forythialan A, phillygenin and phillyrin may be the main active constituents with IC_50_ values of 10.43 ± 0.15, 29.85 ± 0.43, 53.64 ± 2.70, 351.14 ± 13.15 μg/mL, respectively.	[124]
ABTS radical cation	Calceolarioside C	Calceolarioside C scavenged the ABTS radical cation with IC_50_ values of 22.7 μg/mL and the Vc exhibited an IC_50_ of 7.2 μg/mL.	[25]
ABTS radical cation	Lianqiaoxinoside B Forsythoside H	Lianqiaoxinoside B and forsythoside H scavenged the ABTS radical cation with IC_50_ values of 15.6 and 17.7 μg/mL, respectively, while Vc exhibited an IC_50_ of 6.8 μg/mL.	[28]
DPPH, Fe^3+^ and Fe^2+^	Ethyl acetate extract	Ethyl acetate extract (1.0 mg/mL) of *Forsythiae Fructus* exhibited a scavenging rate of 71.39% on the DPPH. It also had a relatively strong ability to reduce Fe^3+^ and chelate Fe^2+^.	[125]
Peroxynitrite-treated LLC-PK1 cell	Phillygenin 8-Hydroxypinoresinol	Phillygenin and 8-hydroxypinoresinol significantly decreased the leakage of lactate dehydrogenase (LDH) at 10 μΜ and even reverse the LDH release induced by 3-morpholinosydnonimine, an ONOO^−^ generator, at 50 μM.	[126]
High-density lipoprotein	Pinoresinol, Phillygenin, 8-Hydroxypinoresinol, 7′-Epi-8-Hydroxypinoresinol, Lariciresinol, Isolariciresinol, Olivil, Cedrusin	The lignans inhibited the generation of thiobarbituric acid-reactive substances in a dose-dependent manner with IC_50_ values from 8.5 to 18.7 μM and thermo-labile radical initiator-induced lipid peroxidation with IC_50_ values from 12.1 to 51.1 μM. Among them, pinoresinol and lariciresinol also exerted an inhibitory effect against Cu^2^+-induced lipid peroxidation of HDL at a concentration of 3 μM.	[32]
D-galactose induced aging mice	Phillyrin	A decrease in weight gain rate, spleen index, SOD, GSH-Px and T-AOC activities in serum and liver tissue and an increase in the content of MDA and MAO-B activities in brain tissue were observed after injection of 15 or 45 mg/kg phillyrin.	[127]
**Antioxidant Activity**			
Weaned piglets	Ethanol extract	Dietary supplementation (100 mg/kg) of *Forsythiae Fructus* ethanol extracts after fourteen days significantly increased glutathione peroxidase activities and serum complement 4 concentration and lowered serum endotoxin and MDA concentration. The oxidative injury disappeared after twenty-eight days.	[128]
Corticosterone-treated broilers	Methanol extract	Dietary supplementation (100 mg/kg) of *Forsythiae Fructus* methanol extract attenuated the decrease of the total antioxidant capacity and SOD activity and increase of serum MDA.	[129]
Arbor Acres broilers under high stocking density	Methanol extract	Treatment with *Forsythiae Fructus* methanol extract (100 mg/kg) increased serum T-AOC and SOD activity and reduced MDA expression. However, no significant differences were found in serum GSH-Px activity.	[130]
**Neuroprotective Activity**			
Rotenone-stimulated PC12 cells and male Sprague-Dawley rats	Ethanol extract	The ethanol extract (50 and 200 mg/kg) exhibited neuroprotective activity by down-regulating protein expression of p-PI3K, p-Akt, p-IκB, p-P65 and cleaving caspase 8, p-p38 and p-JNK.	[131]
SAMP8 mice with composite Alzheimer‘s disease	Forsythoside A	Forsythoside A (60, 120 and 240 mg/kg) increased the activity of SOD, ChAT, and GSH-PX inordinately and decreased the content of MDA and NO by varying degrees in a dose-dependent manner.	[132]
SAMP8 mice	Forsythoside A	Oral administration of forsythoside A (60, 120 and 240 mg/kg) decreased the levels of IL-1β, NO, MDA and NE and increased the T-SOD and GSH-Px activities and the production of GLU and Ach.	[133]
Scopolamine-induced learning and memory impairment in mice	Forsythoside A	Forsythoside A (200 mg/kg) ameliorated scopolamine-induced learning and memory impairment by modulating AchE activity, cAMP expression and p-ERK production and protecting against oxidation.	[134]
Gerbils with transient cerebral global ischemia	Forsythoside A	Oral administration of forsythoside A (10 mg/kg) significantly increased the number of viable neurons and decreased degenerating neurons, activated glial cells and the expression of IL-1β and TNF-α, indicating the involvement of anti-inflammatory activities.	[135]
Aβ_25-35_ oligomer-stimulated HT22 cells	Forsythoside A	Forsythoside A (25 μg/mL) significantly decreased production of NO to improve neuroinflammation in Aβ_25-35_ oligomer-stimulated HT22 cells.	[136]
**Neuroprotective Activity**			
Glutamate or low-glucose and low-serum or Aβ_25-35_-stimulated PC12 cells	Forsythoside A	Forsythoside A (0.1, 1 and 5 μmol/L) improved proliferation of PC12 cells and significantly reduced cell death in vitro. Moreover, forsythoside A (0.1 and 1 μmol/L) significantly inhibited cell apoptosis induced by Aβ_25-35._	[137]
MPP^+^-stimulated SH-SY5Y neuroblastoma cells	Phillyrin	Phillyrin (1, 10 and 100 μmol/L) significantly increased cell viability and reduced leakage of LDH induced by MPP^+^.	[138]
Rotenone-stimulated PC12 cells	Forsythoneoside B Forsythoneoside D	Forsythoneoside B and forsythoneoside D at 0.1 μM inhibited PC12 cell damage induced by rotenone and increased cell viability from 53.9 ± 7.1% to 70.1 ± 4.0% and 67.9 ± 5.2%, respectively.	[11]
**Anti-tumor Activity**			
The murine melanoma B16-F10 cell line and C57BL/6 mice bearing melanoma	Aqueous extract	The aqueous extract inhibited proliferation and angiogenesis of cancer cells, which were closely related to the antioxidant and anti-inflammatory activities via the MAPKs/Nrf2/HO-1 pathway.	[7]
HeLa cells	Aqueous extract	The aqueous extract (50 μg/mL) promoted activation of the zymogen of caspase 8 to inhibit proliferation of cells in vitro time-dose-dependently, with IC_50_ values of 93.74, 33.30 and 22.65 μg/mL for 12, 24 and 48 h, respectively.	[139]
HeLa cells	Ethanol extract	In vitro, the ethanol extract (12.5–100 μg/mL) had an inhibitory effect on the proliferation of Hela cells in a time-dose-dependent manner with IC_50_ values for the 12, 24 and 48 h groups of 97.68, 39.16 and 25.83 μg/mL, respectively.	[140]
SGC7901 cells	Aqueous extract	In vitro, the aqueous extract (25–100 μg/mL) inhibited proliferation of SGC7901 cells in a time-dose-dependent manner with IC_50_ values for the 6, 12 and 24 h of 73.27 ± 3.19, 44.63 ± 2.06 and 35.99 ± 2.43 μg/mL, respectively.	[141]
C57BL/6J mice injected with Lewis cells	Phillyrin	Phillyrin (5 and 10 g/kg) significantly inhibited the tumor size and tumor tissue density dose-dependently by decreasing the expression of VEGF and increasing the expression of endostatin.	[142]
**Anti-tumor Activity**			
A549, Colo205, Hep-3B, HL60, and KB cancer cell lines	(+)-8-Hydroxyepipinoresinol-4-*O*-β-d-glucopyranoside	(+)-8-hydroxyepipinoresinol-4-*O*-β-d-glucopyranoside showed significant cytotoxicity in A549, Colo205, Hep-3B, HL60 and KB cancer cell lines with IC_50_ values of 9.48, 7.75, 0.59, 4.06 and 38.38 μM, respectively.	[34]
MKN-45, MKN-28, SGC-7901, PNAC-1 and HepG-2 cancer cell lines	Ambrolic acid Dammar-24-en-3β-acetoxy-20-ol	Ambrolic acid inhibited SGC-7901 cells by affecting the S period of DNA synthesis and also reduced the levels of pro-caspase 3, 6, 8, 9 and Bcl-2 proteins and increased the levels of Bax protein to induce cell apoptosis, while dammar-24-en-3β-acetoxy-20-ol only had an inhibitory effect on the cancer cells.	[51,55]
PC3 cells of prostate cancer	Dammar-24-en-3β-acetoxy-20-ol	Dammar-24-en-3β-acetoxy-20-ol (6.25–50.0 μg/mL) increased expression of p21, TGF-β and Smad3 and decreased expression of Cyclin D1 and CDC25A to induce cell apoptosis and inhibited the activity of telomerase. Moreover, it affected the radiosensitivity of PC-3 cells of prostate cancer at 25 μg/mL.	[143]
**Hepatoprotective Activity**			
CCl_4_-induced toxicity in rats	Phillygenin	Phillygenin at 0.15 and 0.5 mg/kg significantly decreased the levels of ALT, AST, total bilirubin, TNF-α and IL-8 in serum and the content of MDA in liver tissue. Meanwhile, it increased the activities of SOD, GSH-Px and GSH.	[10]
Bovine serum albumin-induced hepatic fibrosis in rats	Forsythoside A	Forsythoside A alleviated hepatic fibrosis at 0.1, 0.3 and 1.0 mg/kg by decreasing the hydroxyproline content and the levels of layer fibronectin, hyaluronic acid, IV-collagen and procollagen III.	[144]
Human normal liver cell lines LO2	Forsythin	Forsythin reversed nuclear condensation and nuclear fragmentation and decreased expression of apoptosis related proteins (PARP and caspase 3) to prevent alcoholic liver injury does-dependently.	[145]
Rats with severe acute pancreatitis	Aqueous extract	The aqueous extract (1.25, 2.5 and 5 g/kg) significantly reduced the serum levels of amylase, ALT and TNF-α in a dose dependent manner and expression of NF-κB mRNA and Foxp3 mRNA in liver tissue.	[146]
**Cardiovascular Protective Effect**			
Streptozotocin-induced diabetic mice	Ethyl acetate extract	Oral administration of the extract (50, 100 and 200 mg/kg) after four weeks significantly decreased the levels of blood glucose, triglyceride, creatinine and so on and increased body weight, insulin secretion and glucose tolerance, which were related to inhibition of glucokinase, phosphorenolpyruvate carboxykinase, insulin-1, insulin-2 and duodenal homeobox factor-1, thus exhibiting antidiabetic and antihyperlipidemic activities.	[147]
SD rats with atherosclerosis	Phillyrin	Phillyrin (150 mg/kg) reduced the area of AS plaques and the contents of ICAM-1, VACM-1, IL-1, IL-6 and MDA and increased the contents of NO and SOD, probably by decreaseing expression of sodium hydrogen exchange protein 1 (NHE-1).	[12]
Rat aortic rings	Forsythoside A	Forsythoside A inhibited norepinephrine-stimulated vasocontraction by decreasing calcium influx from the extracellular space.	[148]
**Others**			
Cisplatin-treated mice	Aqueous decoction	The aqueous decoction reduced the contents of serum gastrin and promoted gastrointestinal movement at 3, 6 and 12 g/kg, indicating its anti-vomiting activity.	[9]
HepG2 cells	Phillyrin	Phillyrin at the concentration of 1, 2.5 and 5 μM induced phosphorylation of LKB1 and activated AMPK, thus reducing expression of SREBP-1c and fatty acid synthase and avoiding accumulation of lipid.	[149]
TNF-α-stimulated 3T3-L1 adipocytes	Phillyrin	Phillyrin (40 μM) suppressed activation of I kappaB kinase and N-terminal kinase to attenuate TNF-α-mediated insulin resistance and lipolytic acceleration.	[150]
Obese C57BL/6J mice	Phillyrin	Treatment with phillyrin (15 and 45 mg/kg) significantly decreased body weight, the serum levels of TNF-α and leptin and increased expression of PPAR-β/δ, ANGPTL4 and p-AMPK-α.	[151]
Dihydrotestosterone-stimulated mice	Forsythoside A	Forsythoside A suppressed apoptosis of hair cells by reducing expression of caspase-9 by 40%, caspase-3 by 53% and increasing the Bcl-2/Bax ratio by 60%. It also retarded the entry into the catagen phase and reduced the expression of TGF-β2 by 75%.	[152]
Mice with endotoxemia	Forsythoside A	Forsythoside A (80 mg/kg) enhanced the immune function of mice with endotoxemia, which may be associated with the inhibition of TNF-α and IL-10 secretion and the gene expression of Foxp3.	[153]
Yeast-stimulated C57BL/6 mice	Forsythoside A	Forsythoside A (4 and 8 mg/kg) significantly decreased the temperature of mice by up-regulating expression of TRPA_1_ in the paraventricular nuclei (PN), supraoptic nucleus (SO) and dorsal root ganglion (DRG).	[154]
Caco-2 cells	Forsythoside A	Forsythoside A inhibited P-gp ATPase activity to influence the efflux of drugs.	[155]

**Table 4:** The investigations about pharmacokinetics of *Forsythiae Fructus*.

Markers	Methods	Results	Reference
Forsythoside A, Rutin, Phillyrin, Isorhamnetin and Quercetin	HPLC-MS/MS	The *t*_1/2_ of forsythoside A, rutin, phillyrin, quercetin and isorhamnetin after single oral administration of UFF extract were 1.91 ± 1.76 h, 1.59 ± 0.92 h, 3.52 ± 4.37 h, 2.70 ± 2.70 h and 6.32 ± 4.69 h, respectively, while those were 4.52 ± 4.77 h, 6.54 ± 8.73 h, 14.74 ± 27.34 h, not detected and not detected after single oral administration of RFF extract. The *AUC*_0–24 h_ of forsythoside A, rutin and phillyrin were significantly different between sigle oral administration of UFF and RFF extract.	[59]
(+)-Pinoresinol-β-d-glucoside, Matairesinol-4′-*O*-glucoside, Hyperin, Phillyrin, Phillygenin	HPLC-ESI-MS/MS	The average percentages of (+)-pinoresinol-β-d-glucoside, matairesinol-4′-*O*-glucoside, hyperin, phillyrin and hillygenin excreted in the bile over the dose administered (12 mL/kg body weight) were 0.002%, 0.234%, 0.116%, 0.288%, and 12.700%, respectively. Hyperin was found in plasma, urine and excrement of rat while the others were detected only in bile, indicating lignans of *Forsythiae Fructus* were excreted mainly via bile.	[31]
Forsythoside A	LC-MS/MS	Forsythoside A was rapidly absorbed into the blood with a *T_max_* of 20.0 min after oral (100 mg/kg) administration, but the *C_max_* was only 122.2 ± 45.4 ng/mL, indicating a quite low absolute bioavailability with a value of 0.5%.	[158]
Forsythoside A	Microdialysis coupled with HPLC	Forsythoside A went through hepatobiliary excretion and the bile-to-blood distribution ratio (*AUC*_bile_/*AUC*_blood_) was 0.32 ± 0.06 after the intravenous administration of 50 mg/kg.	[159]
Phillyrin	UPLC-Q-TOF-MS	A total of thirty-four metabolites of phillyrin were detected in rat bile, urine and feces and M26 was the major one. Phillyrin mainly went through hydrolysis, oxidation and sulfation to transform into the effective forms in vivo.	[161]
Phillygenin	HPLC	The elimination half-time (t_1/2z_) of phillygenin after intravenous administration of 1.4, 2.8 and 5.6 mg/kg were 6.02 ± 1.66, 5.62 ± 0.35 and 5.79 ± 0.81 min, respectively and the *AUC*_(0–∞)_ were 166.29 ± 18.01, 242.40 ± 7.12 and 332.48 ± 23.98 mg/L min, respectively. All these results suggested the pharmacokinetics of phillygenin followed first-order kinetics.	[162]
Phillyrin and Forsythoside A	UHPLC-MS-MS	The *t*_1/2_ of caffeine, tolbutamide, metoprolol and dapsone in rats after intraperitoneal administration were 5.86 ± 0.83, 5.87 ± 0.83, 4.67 ± 0.63 and 1.17 ± 0.15 h, respectively. But when given a pretreatment of phillyrin and forsythoside A, the *t*_1/2_ of them changed into 4.63 ± 0.56 and 4.15 ± 0.54, 5.56 ± 0.72 and 4.28 ± 0.74, 3.69 ± 0.54 and 4.17 ± 0.27, 1.05 ± 0.15 and 1.02 ± 0.19 h for phillyrin and forsythoside A, respectively, indicating the inductive effect of phillyrin and forsythoside A on CYP. Further study revealed that phillyrin induced rat CYP1A2 and CYP2D1, while forsythoside A induced CYP1A2 and CYP2C11.	[163]
